# Eutectic Coamorphous System of Enzalutamide and Acetyl Maltose: A Strategy for Improved Physical Stability and Aqueous Solubility

**DOI:** 10.1021/acs.molpharmaceut.5c01164

**Published:** 2026-02-23

**Authors:** Julia Cichocka-Łokuciejewska, Justyna Knapik-Kowalczuk, Mateusz Dulski, Katarzyna Ewa Greber, Wiesław Sawicki, Marian Paluch

**Affiliations:** † Institute of Physics, Faculty of Science and Technology, 49568 University of Silesia in Katowice, 75 Pułku Piechoty 1A, Chorzów 41-500, Poland; ‡ Department of Physical Chemistry, Faculty of Pharmacy, Medical University of Gdańsk, Al. Gen. J. Hallera 107, Gdańsk 80-416, Poland; § Institute of Materials Science, Faculty of Science and Technology, University of Silesia in Katowice, 75 Pułku Piechoty 1A, Chorzów 41-500, Poland

**Keywords:** enzalutamide, eutectic composition, co-amorphous systems, physical stability, molecular mobility, aqueous solubility

## Abstract

The low aqueous solubility of crystalline enzalutamide (ENZ) and the limited physical stability of amorphous ENZ present significant formulation challenges. In this study, we explore binary co-amorphous systems of ENZ with octaacetyl maltose (acMAL), focusing on the system having eutectic concentration (ENZ + 75 wt % acMAL) as a potential strategy to enhance both stability and solubility. Based on differential scanning calorimetry (DSC) studies of crystalline materials, the eutectic point was identified, while analysis of DSC thermograms of co-amorphous systems revealed pronounced deviations in values of glass transition temperature (*T*_g_) from Gordon–Taylor predictions, implicating the existence of strong specific intermolecular interactions. FTIR studies confirmed the presence of heteromolecular bonding within the mixtures. Broadband dielectric spectroscopy (BDS) showed that, although acMAL increases ENZ molecular mobility, the eutectic co-amorphous formulation significantly suppresses recrystallization under isothermal conditions (*T* = 413 K), delaying crystallization onset by over 30 h and limiting crystallinity to ≤2% after 55 h. The eutectic ENZ + acMAL composition exhibited sustained supersaturation in both aqueous and biorelevant media, demonstrating a balanced combination of efficient drug release and superior stabilization against recrystallization. These results confirm that eutectic formation followed by co-amorphization of ENZ with acMAL effectively addresses the dual challenges of limited physical stability and poor aqueous solubility. This approach provides a mechanistically rational and transferable strategy for improving the performance of poorly water-soluble APIs in pharmaceutical formulations.

## Graphical abstract

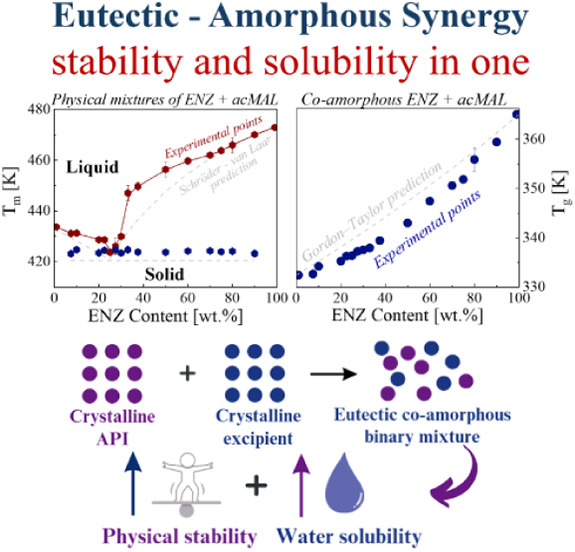

## 1 Introduction

The latest global statistics for 2022 reveal that there were almost 1.46 million newly diagnosed prostate cancer (PC) cases, making it the second most prevalent cancer among men worldwide.^1^ Moreover, it is estimated that by 2050, this number may double.^2^ One of the key drugs in PC therapy is enzalutamide (ENZ), which blocks androgen receptors and inhibits tumor growth, even under conditions of low androgen levels.^3^ The use of ENZ significantly improved survival rates compared to placebo, as demonstrated in the AFFIRM and PREVAIL trials.^4–6^

Despite its therapeutic benefits, ENZ’s lipophilic nature results in low water solubility—a concern shared by approximately 40% of currently available medications and up to 75% of promising drug candidates.^7^ Existing ENZ formulations, marketed as Xtandi Tablets, were developed and introduced in 2020 by Medivation Inc. and Astellas Pharmaceutical Inc. and are available as 40 mg tablets (10.1 mm in diameter) and 80 mg tablets (17.2 × 9.1 mm).^8^ This means that to achieve an effective dosage of 160 mg, patients must take two or four relatively large tablets, potentially complicating adherence to treatment protocols, especially among older men who constitute the majority of prostate cancer patients.^9^ Large tablets may be difficult to swallow, impacting treatment consistency.^10^ Therefore, improving ENZ’s solubility is crucial, as it could enable lower-dose regimens, potentially minimize side effects, enhance the drug’s overall safety profile, and facilitate medication management for patients.

The Biopharmaceutical Classification System (BCS) categorizes drugs based on their solubility and permeability, emphasizing that compounds like ENZ, which have low water solubility, encounter significant challenges during pharmaceutical development.^3,11^ Consequently, strategies aimed at increasing the dissolution rate are crucial for optimizing the therapeutic effectiveness and ensuring patient compliance. As a result, numerous researchers globally are investigating novel methods and improving well-known ways to enhance the solubility of poorly soluble drugs. These include chemical modifications, specifically the formation of salts, which can significantly improve solubility in the gastrointestinal environment.^12^ Another technique is microencapsulation, where the active substance is enveloped by a carrier material, enhancing stability and dissolution.^13^ Additionally, the amorphous solid dispersions (ASDs), in which the active pharmaceutical ingredient (API) is dispersed in a carrier in its amorphous state, can also result in a solid form with significantly greater solubility compared to its crystalline counterpart.^14,15^

In the case of ENZ, the use of ASD has shown promising results; however, there is still room for improvement. The use of polymers significantly increases the tablet mass. Additionally, amorphous materials often suffer from limited physical stability due to thermodynamically metastable or unstable states, which drive them to recrystallize. This tendency presents a significant challenge for maintaining the improved solubility benefits that ASDs initially offer.^16^

So far, studies have mainly focused on creating ASDs with polymers. The key element is the selection of appropriate polymers, which should be biocompatible and safe for the patient. Cellulose derivatives are of great interest due to their properties, and many of them have been approved by the Food and Drug Administration (FDA) for pharmaceutical use.^17,18^ Nevertheless, the effectiveness of ASDs depends largely on the structure of the polymer and the interaction with the API. Hydrophobic polymers effectively inhibit crystallization, while hydrophilic ones promote API solubility.^19^ However, despite the advantages of the development of ASDs, challenges remain. Issues such as the lack of stability of amorphous forms, the risk of recrystallization, and the need to balance hydrophilic and hydrophobic properties require further research. Not all FDA-approved polymers are effective in creating stable ASDs, highlighting the need for innovative approaches to enhance drug solubility and bioavailability while ensuring patient safety.^20–22^ This indicates a clear gap that researchers continue to explore for improved formulations.

To address this issue, coamorphous binary mixtures of APIs and low molecular weight excipients are being considered.^23^ The goal of the current study was to create a co-amorphous eutectic composition with ENZ and acetylated maltose (acMAL). Eutectic systems promote a co-amorphous state by simultaneously lowering the melting point of two combined components compared to their individual melting temperatures. Moreover, some studies suggest that eutectic systems can enhance the dissolution rate of poorly water-soluble drugs, like ENZ, improving their bioavailability.^24–26^ In this study, we will verify the hypothesis that a co-amorphous system having eutectic concentration based on ENZ is characterized by the highest and sustained supersaturation and the longest physical stability among other concentrations of these compositions.

## 2 Experimental Methods

### 2.1 Pure Compounds and Binary Mixtures

Enzalutamide (ENZ) (C_21_H_16_F_4_N_4_O_2_S, *M*_w_ = 464.436 g/mol, Lot: P180-02100, purity better than 95%) was purchased from AstaTech and employed in its original state without any additional purification. β-D-Maltose octaacetate (acMAL) (C_28_H_38_O_19_, *M*_w_ = 678.59 g/mol, Lot: 1301) was obtained from Iris Biotech GmbH. The molecular structures of both molecules are shown in Figure 1. Hydroxypropyl methylcellulose acetate succinate (HPMCAS, AQOAT AS-MF, Lot: 104035) was supplied by Shin-Etsu Chemical Co., Ltd. and used as received. FaSSIF v2 (fasted state simulated intestinal fluid, version 2) was prepared and used according to the manufacturer’s instructions (Biorelevant.com) and equilibrated for 2 h at 310.15 K prior to use.

**Figure 1:**
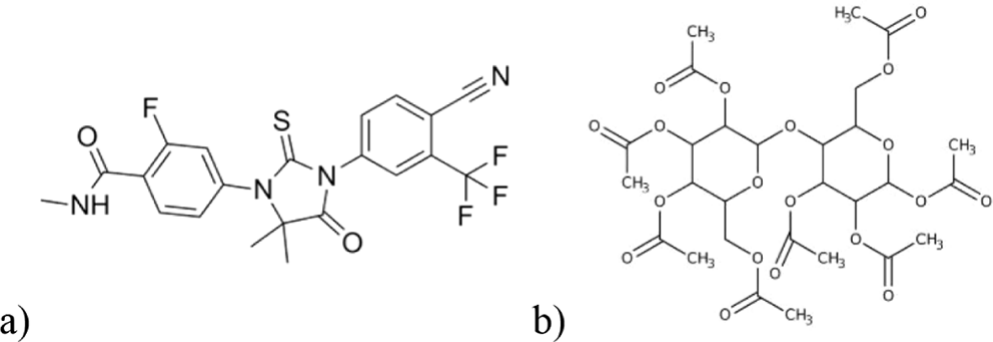
Chemical structures of (a) enzalutamide and (b) β-D-maltose octaacetate.

Binary mixtures were obtained by mixing neat substances in crystalline form using a mortar and grinding them gently for 10 min at room temperature. The concentration of ENZ in the compositions varied from 7.5% to 90% by weight. To achieve amorphous mixtures, the prepared powder blends were heated above their melting point and subsequently cooled to a glassy state. In the case of dielectric measurements, the samples were cooled to 173 K, while for calorimetry measurements, a cooling temperature of 303 K was employed.

### 2.2 Differential Scanning Calorimetry Characterization

The thermal properties of neat, both crystalline and amorphous ENZ and acMAL, as well as their binary mixtures, were examined using a Discovery DSC 250 system manufactured by TA Instruments Inc. For this purpose, powder samples of mass between 3 and 11 mg, either of a neat API or mechanical binary mixtures, were placed into aluminum crucibles (40 μL). As a reference, an empty aluminum crucible was utilized. The proper temperature was obtained by controlled nitrogen gas flow rates (50 mL/min, respectively). Instrument calibration for temperature and enthalpy was performed by using indium and zinc standards. The melting point of the neat substance and the solidus transition in the binary mixture were determined as the onset of the peak, whereas in the case of the liquidus transition, the peak maximum was detected. Glass transition temperature (*T*_g_) was identified as the midpoint of the heat capacity increment. All samples were measured with a heating rate of 10 K/min, while the cooling rate was 20 K/min. Each experiment was conducted at least in triplicate.

### 2.3 Broadband Dielectric Spectroscopy Characterization

The molecular dynamics of amorphous neat ENZ and selected binary amorphous mixtures of ENZ and acMAL were evaluated by using a Novocontrol GMBH Alpha dielectric spectrometer. Experiments were performed in the frequency range from 10^–1^ Hz to 10^7^ Hz and the temperature range from 173 to 433 K with steps of 2 K/min. Temperature control was maintained using a Quatro temperature controller, ensuring stability better than 0.1 K. All samples were measured in a parallel-plate cell made of stainless steel featuring a 15 mm diameter and a 0.1 mm gap with silica spacers. Isothermal crystallization kinetics studies were conducted in a frequency range from 10^1^ Hz to 10^7^ Hz at 413 K for neat amorphous ENZ, ENZ + 50 wt % acMAL, and ENZ + 75 wt % acMAL.

### 2.4 Fourier Transform Infrared Spectroscopy (FTIR)

Infrared spectroscopy for neat ENZ and acMAL, as well as their binary mixture with different concentrations of ENZ-to-acMAL, was carried out using an Agilent Cary 640 FTIR spectrometer equipped with a standard source and a DTGS Peltier-cooled detector. Spectra were collected over the 400–4000 cm^–1^ range using a GladiATR diamond attenuated total reflectance (ATR) accessory. Temperature-dependent studies were carried out by using a heated GladiATR module at a heating rate of 10 K/min, with spectra recorded at each temperature point. Each spectrum was averaged from 16 scans at a resolution of 4 cm^–1^. Postacquisition processing was performed using Win-IR Pro (v2.96), including baseline correction, atmospheric water and CO_2_ removal, and ATR correction based on the refractive indices of the individual APIs (*n* = 1.629 for ENZ and *n* = 1.507 for acMAL).

### 2.5 High-Performance Liquid Chromatography

#### 2.5.1 Powder Dissolution Studies in FaSSIF

Powder dissolution studies of amorphous ENZ and its binary mixtures with acMAL were carried out under non-sink conditions in a biorelevant medium. Each formulation was tested as a 300 mg powder dose. All formulations contained a constant 40 wt % HPMCAS, while the ENZ-to-acMAL ratio varied as specified in Table 2. HPMCAS was blended with the respective ENZ/acMAL physical mixtures, melt-processed, cooled to room temperature, and subsequently ground and sieved to obtain particle size fractions between 100 and 200 μm prior to dissolution testing. The experiments were performed in FaSSIF (pH 6.5) using a dissolution apparatus (Erweka DT 800) filled with 500 mL of medium, maintained at 310.15 ± 0.5 K, with the paddle speed set to 50 rpm. The dissolution rate was measured at time points of 1, 2, 3, 5, 10, 15, 20, 25, 30, 35, 40, 45, 50, 55, 60, 90, 120, 150, 180, 210, and 240 min. Each time, 3 mL of sample was taken, and the medium was replenished to the initial volume so as not to disturb the solubility test conditions. The samples were filtered immediately through a 0.45 μm syringe filter and then diluted 2-fold with acetonitrile and quantified using HPLC as described in Section 2.5.2. All measurements were performed in triplicate.

#### 2.5.2 HPLC Analysis

The resulting solutions of ENZ and ENZ systems were subjected to chromatographic analysis using an HPLC system (Shimadzu Prominence-i LC-2030C 3D) equipped with a UV detector. A 20 μL sample was injected into a Macherey-Nagel Nucleoshell C18 column (150 mm × 4.6 mm, 5 μm) maintained at 303.15 K. The separation was performed using a mobile phase of acetonitrile and water (55:45, v/v) at a 1.0 mL/min flow rate, with detection at 239 nm.^27^ To determine the concentration of dissolved API, the obtained chromatograms were analyzed for the area under the peak and compared against a previously established calibration curve, validated for specificity, linearity, precision, accuracy, limit of detection, and limit of quantification, following the International Council for Harmonisation Q2­(R1) guidelines. Each experiment was conducted in triplicate.

## 3 Results and Discussion

### 3.1 Characterization of Neat Crystalline and Amorphous Enzalutamide

At the initial stage of neat Enzalutamide (ENZ) characterization, differential scanning calorimetry (DSC) was employed to determine its melting point (*T*_m_) and glass transition temperature (*T*_g_). In the DSC thermogram of crystalline ENZ (Figure 2, solid blue line), a sharp endothermic peak with an onset at 473 K indicated the substance’s melting. To obtain amorphous ENZ, the sample was cooled to room temperature at 20 K/min and then reheated. The resulting thermogram (solid gray line, Figure 2) showed a step-like transition with a midpoint at *T*_g_ = 364 K. This relatively high *T*_g_ suggests that amorphous ENZ is likely to exhibit high physical stability during storage at room temperature due to restricted molecular mobility. Literature indicates that when storage temperature is at least 50 K below *T*_g_ (over 60 K for ENZ), molecular motion is sufficiently restricted to effectively inhibit crystallization during storage.^28,29^

**Figure 2:**
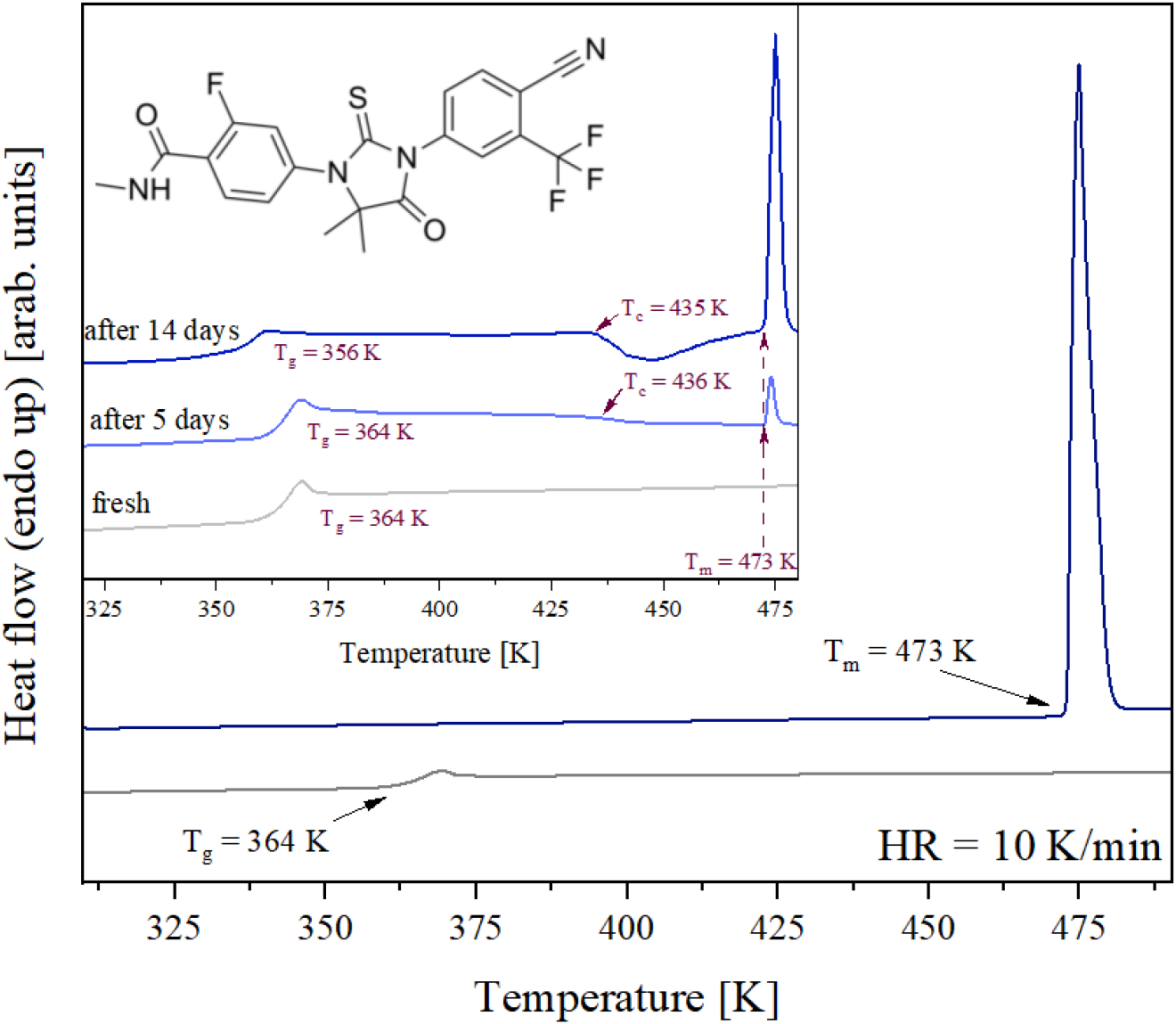
DSC thermograms of neat crystalline and amorphous forms of ENZ. The inset shows a thermal comparison of crystalline and amorphous forms of ENZ after 5 and 14 days of storage at room temperature.

To verify this hypothesis and to assess the preliminary stability of the amorphous form of ENZ, DSC thermograms were recorded after 5 and 14 days of storage at room temperature (see inset in Figure 2). The 5-day sample still shows a clear glass transition at 364 K, identical to freshly amorphized ENZ. Upon further heating, a weak exothermic peak appears, indicating recrystallization, followed by melting at 436 K. This effect intensifies with time: after 14 days, a stronger recrystallization exotherm and melting peak are observed. The glass transition also shifts to a lower temperature of 356 K, likely due to moisture uptake, which acts as a plasticizer and lowers *T*_g_, as reported in various studies.^30^

These results suggest that although amorphous ENZ initially exhibits good physical stability, prolonged storage at room temperature can initiate nucleation processes even below *T*_g_. Literature reports indicate that nucleation in amorphous drugs can occur well below the glass transition temperature, with the number of nuclei accumulating over time despite limited molecular mobility.^31^ This accumulation increases the likelihood of crystallization during subsequent heating, storage, or manufacturing. For instance, an elevated pressure applied during tableting may trigger recrystallization in such a material, thereby diminishing the physical robustness of the amorphous state.

Following thermal characterization, broadband dielectric spectroscopy (BDS) was employed to investigate the molecular dynamics of amorphous ENZ in greater detail. Such analysis is crucial for evaluating the sample relaxation processes below and above *T*_g_, which directly influence the material’s long-term physical stability. Representative dielectric loss spectra are shown in Figure 3, with panel (a) corresponding to temperatures above *T*_g_ and panel (b) corresponding to those below *T*_g_.

**Figure 3:**
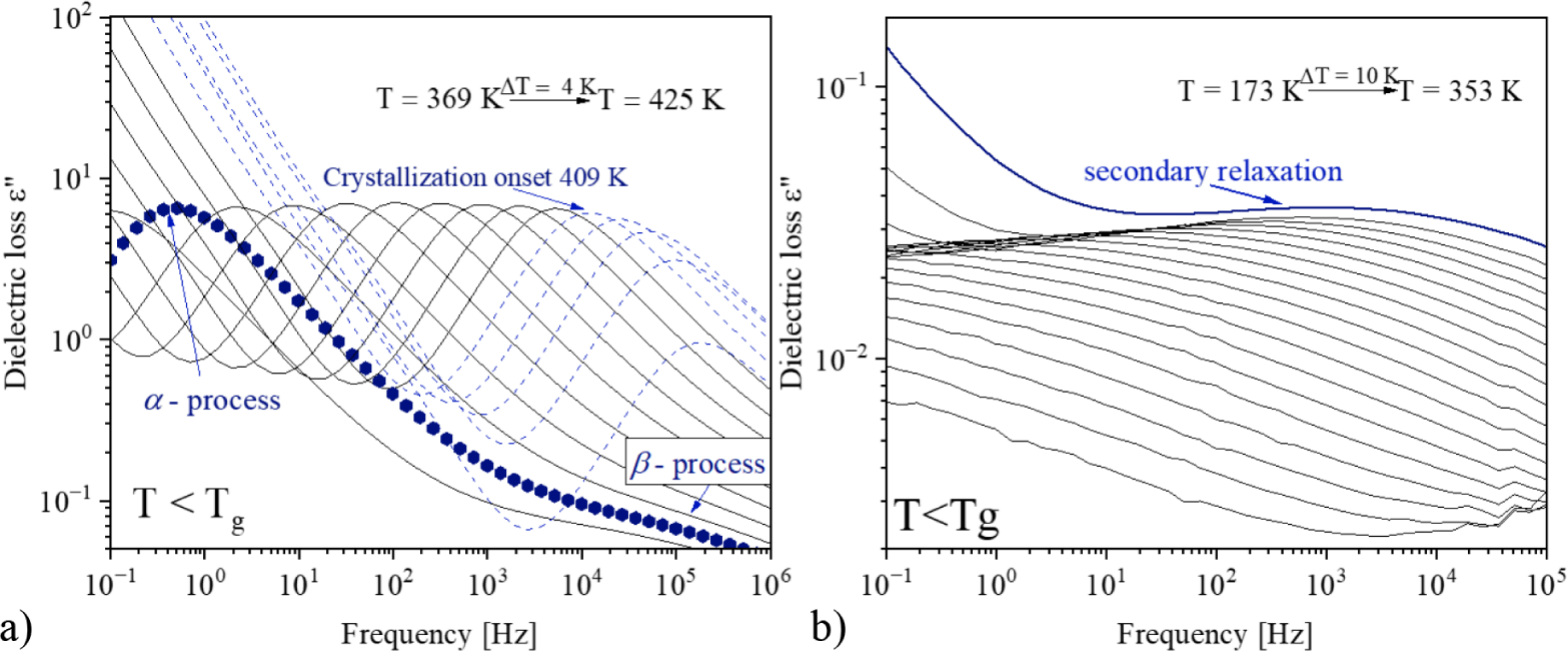
Dielectric loss spectra of neat ENZ obtained on heating. Panel (a) presents spectra collected above the glass transition temperature, whereas panel (b) shows spectra below *T*_g_.

The dielectric loss spectra recorded in the supercooled liquid state show a distinct α-relaxation peak corresponding to the global molecular motions of ENZ. Near the glass transition temperature, where the α-process maximum appears between 10^–1^ and 10^1^ Hz, a weaker β-process can also be seen on the high-frequency side of the spectrum. The β-process has a significantly lower intensity due to its local nature, unlike the cooperative dynamics of α-relaxation. As temperature increases, both relaxation bands shift toward higher frequencies, reflecting enhanced molecular mobility.^32^ At 409 K, a sudden drop in the α-peak intensity marks the onset of cold crystallization (panel (a), blue dashed lines). Below *T*_g_, only the β-process remains visible.

For further analysis, a relaxation map was constructed and is presented in Figure 4, showing the relationship between the relaxation times of the α-process and the secondary relaxation β-process of ENZ. In order to determine τ_α_, the asymmetric α-relaxation peaks were fitted using the Havriliak–Negami (HN) function, defined as^33^

1 $$\varepsilon_{\mathrm{HN}}^{*}(\omega )=\varepsilon^{\prime }\left(\omega \right)-i\varepsilon^{\prime \prime }\left(\omega \right)=\varepsilon_{\infty }+\frac{\Delta \varepsilon }{{[1+{\left(i\omega \tau_{HN}\right)}^{\alpha }]}^{\beta }}$$

where ε′(ω) and ε″(ω) are the dielectric responses in real and imaginary parts, respectively, ε_∞_ is the permittivity at high frequencies, while Δε is dielectric strength. Relaxation time, characteristic of the process, is represented by τ_HN_. α and β are peak shape parameters. In the case of secondary relaxation processes, the Cole–Cole function was indicated, which is equivalent to the HN function with β = 1. Then, based on the fitting parameters, τ_α_ and *τ*_β_ were calculated using the equation:

2 $$\tau_{\alpha /\beta }=\tau_{HN}{[\sin\left(\frac{\pi \alpha }{2+2\beta }\right)]}^{-1/\alpha }{[\sin\left(\frac{\pi \alpha \beta }{2+2\beta }\right)]}^{1/\alpha }$$

**Figure 4:**
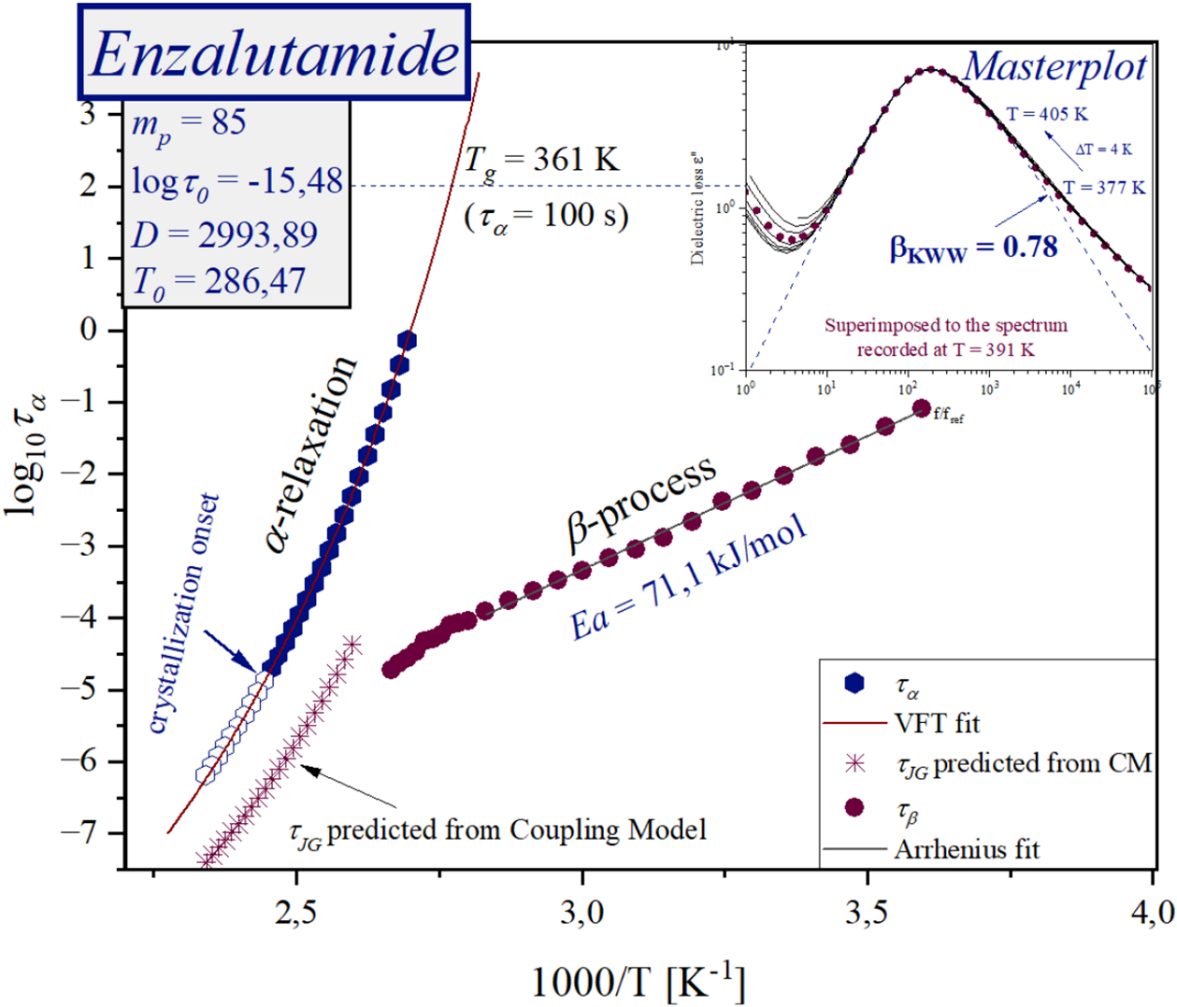
Relaxation map of neat ENZ. The temperature dependence of τ_α_ in the supercooled liquid was described by the VFT equation. The masterplot obtained from spectra recorded at temperatures 377–405 K is shown in the upper right corner.

It is worth pointing out that the values of τ_α/β_ calculated from the above equation correspond to the inverse of the peak frequency (τ_α/β_ = 1/2π*f*_max_), where *f*_max_ denotes the frequency at which the maximum of the respective relaxation process occurs.

To parametrize the temperature dependence of the α-relaxation process, the Vogel–Fulcher–Tammann (VFT) equation was used, which describes the relaxation time as a function of temperature. The VFT relation is given by the equation:^34,35^

3 $$\tau_{\alpha }\left(T\right)=\tau_{\infty }\exp\left(\frac{B}{T-T_{0}}\right)$$

where τ_∞_ means pre-exponential relaxation time, *B* = *DT*_0_, with *D* being the parameter that quantifies the deviation from the Arrhenius behavior, and *T*_0_ corresponds to the temperature at which the relaxation time approaches infinity.

Based on the VFT parameters, the *T*_g_ was determined to be 361 K (defined at τ_α_ = 100 s), which is in good agreement with the results obtained from calorimetric studies (see Figure 2).^36^

Estimating the physical stability of an amorphous drug candidate at an early stage of development is crucial for successful formulation in the pharmaceutical industry. Many studies have aimed to predict the recrystallization tendency of amorphous drugs. However, individual studies are often insufficient to conclusively validate the results, as the physical stability of amorphous materials depends on multiple factors. Therefore, reliable predictions require a comprehensive and multifactorial approach. Although various theories and methods have been proposed, there is still not enough data to develop a universal model capable of effectively predicting the physical stability of amorphous APIs.^37,38^

Among molecular dynamics-based parameters, fragility (*m*_*p*_) is one of the most widely used. It describes the temperature dependence of structural relaxation near *T*_g_ and quantifies the deviation from the Arrhenius behavior:

4 $$m_{p}={\frac{d\log\tau_{\alpha }}{d\left(T/T_{g}\right)}|}_{T=T_{g}}$$

The literature indicates a correlation between physical stability and the value of this parameter, since a higher fragility (*m*_*p*_) implies greater susceptibility of the material to changes in molecular dynamics, which in turn leads to reduced physical stability.^39^ It is also supported by a theoretical model, the two-order parameter (TOP) model proposed by Tanaka.^40^ Typical *m*_*p*_ values range from 16 to 200. For ENZ, a relatively high value of this parameter (*m*_*p*_ = 85) suggests low physical stability, as confirmed by calorimetric and dielectric studies.^36,41^ The same value has been reported for sildenafil, an API that also exhibits a pronounced tendency toward cold crystallization.^42^

Another parameter proposed for predicting the physical stability of amorphous substances is based on the distribution of the Debye relaxation processes.^43^ The β_KWW_ parameter, derived from the Kohlrausch–Williams–Watts (KWW) function, is used to assess the relaxation time distribution of ENZ and to verify whether this distribution remains constant over the examined temperature range.^44^ To investigate this, a so-called masterplot was constructed, as shown in the inset of Figure 4. The masterplot was generated from spectra registered at temperatures ranging from 377 to 405 K in 4 K intervals and superimposed by horizontal shifting onto the reference spectrum obtained at 391 K. This procedure results in a β_KWW_ value equal to 0.78 for ENZ. Values near 1 indicate narrow, symmetrical peaks (homogeneous mobility, higher stability), while smaller values reflect broad, asymmetric peaks, heterogeneous relaxation, and reduced stability (i.e., an increased risk of recrystallization). The β_KWW_ = 0.78 for ENZ implies relatively good physical stability, though experimental data show notable nucleation even in the glassy state. Recent reports have questioned β_KWW_ as a stand-alone predictor of physical stability, since materials with similar β_KWW_ values can exhibit different crystallization behavior.^45^ These inconsistencies highlight the limitations of this parameter when used alone. It is worth noting that, according to the established anticorrelation between the width of the α-loss peak and polarity of the molecule by Paluch et al., glass formers with a broad α-loss peak (i.e., a small value of β_KWW_) should exhibit a low value of the dielectric strength (Δε_α_).^46^ ENZ with β_KWW_ = 0.78 and Δε_α_ = 20, follows this anticorrelation similarly to other APIs like bicalutamide (β_KWW_ = 0.85, Δε_α_ = 60), sildenafil (β_KWW_ = 0.68, Δε_α_ = 8), and chloramphenicol (β_KWW_ = 0.8, Δε_α_ = 55).

After a detailed discussion of the primary relaxation of ENZ, it is worth turning attention to the secondary relaxation, which offers complementary insight into the molecular dynamics of the examined API. While α-relaxation reflects the macroscopic structural dynamics of the amorphous material, the β-process represents more local molecular motions. This aspect is particularly relevant, since under standard storage conditions (i.e., deep in the glassy state, at room temperature), such local secondary motion becomes the dominant form of molecular mobility in amorphous ENZ. Notably, previous studies have shown that secondary relaxations can significantly influence physical stability. For instance, in the case of celecoxib, it has been demonstrated that β-relaxation may play a key role in initiating recrystallization processes, even when the material remains below its glass transition temperature.^47,48^ Thus, in the case of ENZ, the analysis of the secondary relaxation seems crucial for a better understanding of the physical stability of this amorphous API.^49^ As shown in Figure 4, the temperature evolution of ENZ’s secondary relaxation times (τ_β_) in the glassy state displays a linear behavior, which can be accurately described by the Arrhenius equation:^50^

5 $$\tau \left(T\right)=\tau_{0}\exp\left(\frac{E_{a}}{RT}\right)$$

where τ_0_ is the pre-exponential relaxation time, *E*_a_ represents the activation energy, and *R* is a gas constant. It is important to emphasize that secondary relaxations can have different molecular origins and are commonly classified into two types: intramolecular and intermolecular secondary relaxations. Intramolecular relaxations—often referred to as non-Johari–Goldstein (non-JG) processes—are associated with the motion of only a part of the molecule, such as side chains or functional groups. In contrast, intermolecular, i.e., Johari–Goldstein (JG), relaxations come from local motions of the entire molecule. The latter are of particular interest, as they are considered potential precursors to the α-relaxation and are thought to play a crucial role in the recrystallization of amorphous pharmaceuticals. Therefore, from the perspective of physical stability, it is essential to determine whether the β-relaxation observed in ENZ corresponds to a JG process. To confirm this, usually the Coupling Model is employed using the criteria proposed by Ngai and Paluch, as described below:^51^

6 $$\tau_{JG}\approx \tau_{0}={\left(t_{c}\right)}^{1-\beta_{KWW}}{\left(\tau_{\alpha }\right)}^{\beta_{KWW}}$$

In the above equation, τ_0_ is the primitive relaxation time, and *t*_*c*_ corresponds to the onset of intermolecular coupling, which, for small molecules, is equal to 2 ps. The τ_JG_ values of ENZ obtained in this way are presented in Figure 4 as scarlet stars. As can be seen, the values of τ_JG_ estimated based on the applied model suggest an intermolecular origin of the β-relaxation observed in ENZ. These predictions, however, are not conclusive and thus cannot definitively confirm the nature of this relaxation process. To clarify its origin, additional studies—particularly those assessing its sensitivity to elevated pressure—would be highly valuable.

### 3.2 Thermal Characterization of Crystalline and Amorphous Enzalutamide + Octaacetyl Maltose Binary Mixtures

After testing the physical instability of pure amorphous ENZ upon heating, it is necessary to investigate whether the use of a small-molecular excipient could enhance the physical stability of the tested API. Co-amorphization is one of the well-established strategies for suppressing the recrystallization tendency of glass formers.^52^ For this purpose, octaacetyl maltose (acMAL) was selected as a model excipient for ENZ. Although acMAL is not listed in the FDA Inactive Ingredient Database, it has been investigated in recent studies as a stabilizing additive for amorphous pharmaceuticals (e.g., celecoxib, ibuprofen, indomethacin).^53,54^ Its molecular structure and physicochemical properties are analogous to those of other acetylated carbohydrate derivatives, such as sucrose octaacetate and cellulose acetate, which are established as safe excipients. Since acMAL is additionally characterized by good water solubility, the potential to enhance wettability and dispersion, and the ability to stabilize the amorphous form, it was selected in this study as a model and promising pharmaceutical additive. To further maximize its potential, coamorphization at the eutectic composition is proposed, as eutectic mixtures are also known to improve solubility. Therefore, we hypothesize that combining these two strategies—formulating a eutectic system followed by its amorphization—may yield optimal results in terms of enhanced solubility of ENZ in the presence of acMAL, while simultaneously improving the physical stability of the amorphous API through favorable molecular interactions specific to the eutectic composition.^55^

To theoretically determine the concentration at which the eutectic mixture is formed, the Schröder-van Laar equation was applied:^56^

7 $$\ln\left(x\right)=\frac{\Delta H_{0}}{R}\left(\frac{1}{T_{0}}-\frac{1}{T}\right)$$

where *x* represents the molar fraction of the given substance at a melting point equal to *T*, Δ*H*_0_ signifies the enthalpy of fusion (J·mol^–1^), *R* represents the gas constant, and *T*_0_ is the melting point of the pure component. The resulting plots are shown as dashed lines in Figure 5b, representing the liquidus curves. According to this graph, the eutectic composition was estimated at 25 wt % of ENZ and 75 wt % of acMAL.

**Figure 5:**
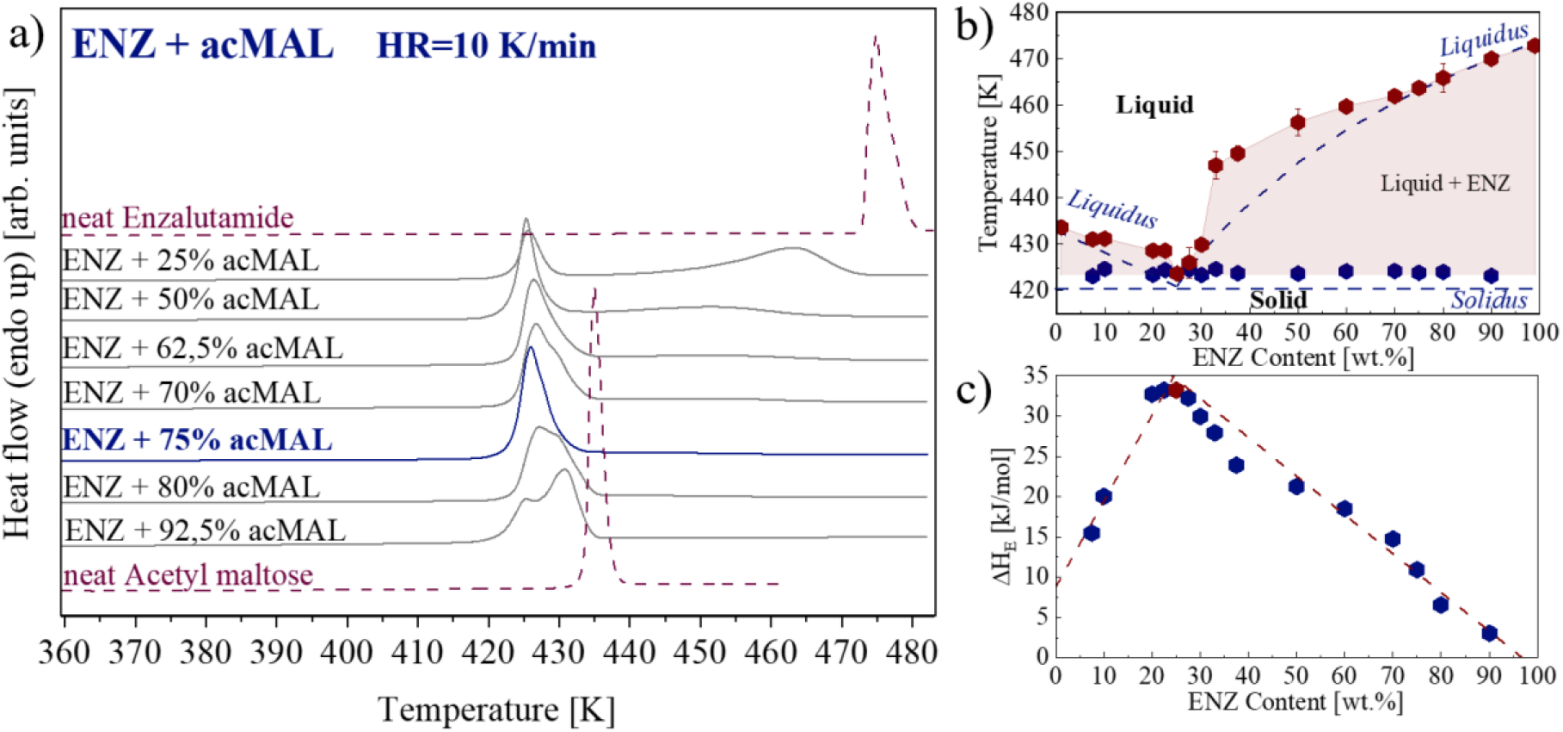
Panel (a) shows thermograms of crystalline neat substances and their binary mixtures, while panel (b) presents the equilibrium phase diagram of the binary ENZ-acMAL system as a function of the ENZ content [wt %]. The panel shows both the experimental data obtained from DSC measurements and the liquidus temperatures predicted with the Schröder-van Laar equation. Panel (c) displays the Tammann plot.

To confirm the predicted eutectic point, binary mixtures with varying acMAL concentrations were prepared, as described in Section 2. All samples were measured using DSC at a heating rate of 10 K/min over the temperature range of 303–483 K. The resulting thermograms of both neat components and their crystalline mixtures are presented in panel a of Figure 5. As can be seen, most binary systems exhibit two distinct endothermic events, corresponding to the melting of each component. In all mixtures, a depression of the melting point of the parent materials is observed. Notably, the sample containing 75 wt % of acMAL displays a single, sharp endothermic peak, indicating the eutectic melting. This observation is consistent with the theoretical prediction based on the Schröder-van Laar equation (Eq 7). To construct the experimental phase diagram, the onset temperature of the first melting event (i.e., located at a lower temperature) and the peak maximum of the second melting endotherm were determined and are plotted as red points in Figure 5b.

As shown in Figure 5b, the experimentally obtained phase diagram does not fully align with the theoretical equilibrium model. The experimental solidus line is shifted toward higher temperatures, which may reflect a higher degree of system complexity, potentially due to the non-ideal mixing behavior of the components. The experimental liquidus temperatures also deviate from those predicted theoretically, suggesting that the observed melting point depression of the parent components may be influenced by the formation of intermolecular interactions between them.^57^

The Schröder-van Laar equation has been proven to be a useful first approximation for estimating the eutectic composition under the assumption of ideal mixing behavior in the liquid phase. However, its predictive power for peak positions is limited. The observed discrepancies between the theoretical and experimental phase behavior of ENZ + acMAL likely arise from the fact that this model does not account for factors such as specific intermolecular interactions between parent compounds or asymmetry in their miscibility, which can significantly influence the phase boundaries of real systems. The eutectic ENZ + acMAL composition showed the most sustained supersaturation in aqueous and biorelevant media, suggesting improved dissolution stability and the potential for enhanced absorption. All these findings demonstrate that eutectic formation followed by co-amorphization of ENZ with acMAL can synergistically address both the physical stability of the amorphous form and the solubility limitations of the crystalline form of poorly water-soluble APIs such as ENZ, offering a promising formulation strategy for pharmaceutical development.

The quench-cooled in situ DSC materials were subsequently reheated (HR = 10 K/min), and the obtained thermograms for both neat amorphous substances and their binary mixtures are presented in Figure 6a. The presence of a glass transition event, together with the absence of a melting endotherm, confirms the amorphous form of the samples. As shown, both the individual substances and their co-amorphous mixtures exhibit a single glass transition event. This suggests the formation of a homogeneous amorphous phase and good miscibility between the components, resulting in a unified structure without signs of phase separation.^58^ In the next step, the experimentally determined *T*_g_ values for the investigated systems were plotted as a function of composition, as presented in panel b of Figure 6. In this plot, the obtained *T*_g_ values were also compared with theoretically predicted *T*_g_ values of the binary systems calculated using the Gordon–Taylor (GT) equation:^59,60^

8 $$T_{g}=\frac{w_{1}T_{g1}+Kw_{2}T_{g2}}{w_{1}+Kw_{2}}$$

where *T*_g_ is the predicted glass transition temperature of the mixture, *w*_1_ and *w*_2_ are the weight fractions of components 1 and 2, respectively, and *T*_*g*1_, *T*_*g*2_ represent the glass transition temperatures of the neat substances. The parameter *K* compensates for differences in the heat capacity change at the glass transition and was calculated as follows:

9 $$K\approx \frac{\Delta C_{p2}}{\Delta C_{p1}}$$

where Δ*C*_*p*1_ and Δ*C*_*p*2_ are the changes in heat capacity at *T*_g_ for neat components.

**Figure 6:**
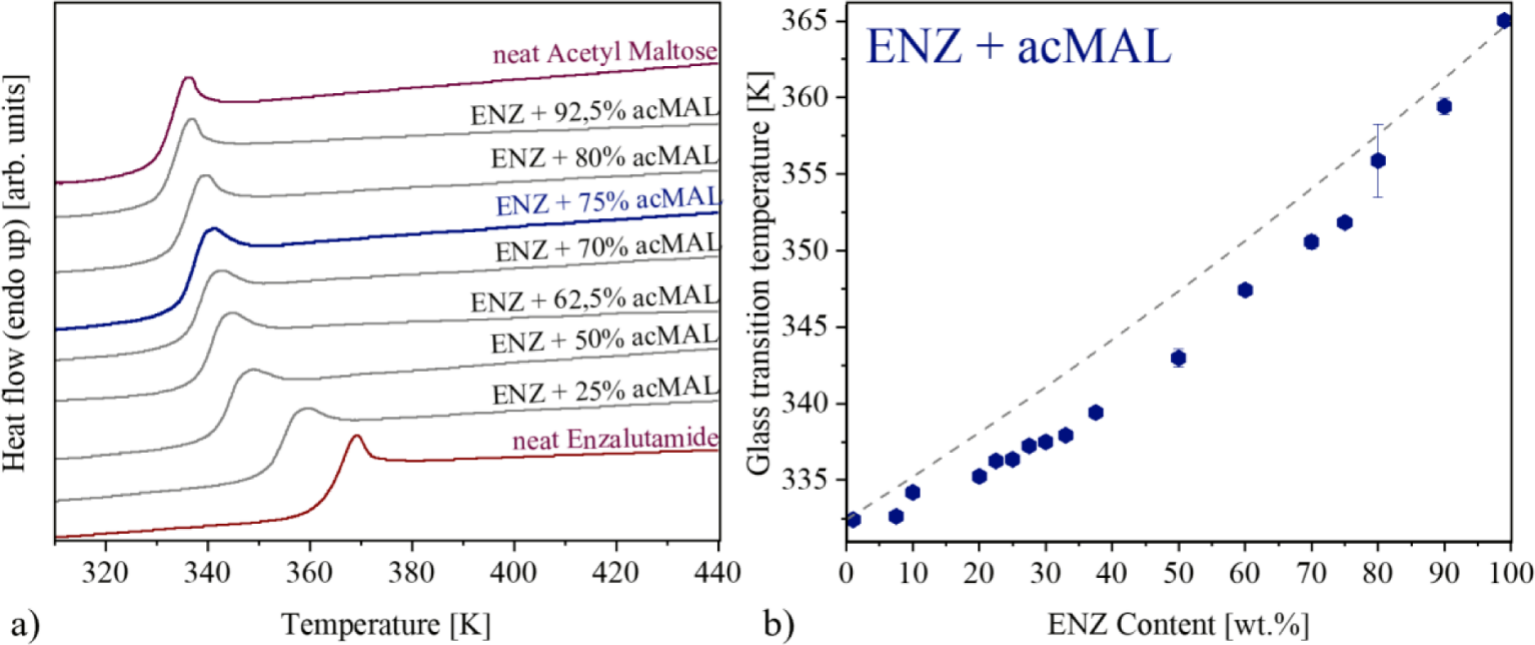
Panel (a) presents DSC thermograms of neat substances and their binary mixtures in amorphous forms (HR = 10 K/min). Panel (b) shows the prediction of the Gordon–Taylor equation calculated from the *T*_g_ values of the single ENZ and acMAL (dashed line). Blue points represent experimentally obtained *T*_g_ values of co-amorphous mixtures. The chemical structures of ENZ and acMAL are also presented in the inset.

Since the GT model predicts the glass transition temperature of an ideal, non-interacting mixture, any deviation of the experimentally determined *T*_g_ values from the theoretical curve suggests the presence of specific intermolecular interactions, particularly hydrogen bonding.^61–63^ As shown in Figure 6b, the experimental data exhibit substantial deviation from the GT prediction (gray dashed line) across a broad concentration range. Notably, the compositions showing the greatest divergence from the model also correspond to those for which the experimental liquidus line of the crystalline counterparts deviates most significantly from the Schröder-van Laar equation, further supporting the hypothesis that the strongest specific interactions between ENZ and acMAL occur in the range of 30–60 wt % of ENZ.

### 3.3 Investigation of Intermolecular Interactions in ENZ + acMAL Mixtures Using FTIR Spectroscopy

To verify whether our hypothesis regarding the presence of intermolecular interactions in the ENZ + acMAL system is justified—and to evaluate both their type and relative strength—Fourier-transform infrared (FTIR) spectroscopy was employed. The study was carried out for both the physical mixtures of crystalline components and the corresponding co-amorphous systems. Four representative compositions of ENZ and acMAL were selected for this analysis. These included the eutectic composition containing ENZ + 75 wt % of acMAL, as well as systems representing ENZ-poor (90 wt % acMAL) and ENZ-rich (40 wt % acMAL) mixtures to explore the effect of varying component ratios. Additionally, a mixture with 50 wt % acMAL was chosen due to the most significant deviation of the experimentally determined *T*_g_ from the value predicted by the Gordon–Taylor model, strongly suggesting the presence of specific intermolecular interactions. To provide a baseline for interpretation, neat ENZ and acMAL were also analyzed as reference materials.

The FTIR studies were divided into two complementary stages. In the first stage, crystalline materials—including both the neat substances and the investigated binary mixtures—were heated at a constant rate of 10 K/min from *T* = 303 K to *T* = 483 K. While in the second stage, the samples were investigated at *T* = 298 K, immediately after cooling from the molten state with a controlled cooling rate of 5 K/min. The temperature-dependent infrared spectra obtained from the first stage allowed for monitoring of structural and chemical changes as a function of the temperature. An example of such data is presented in Figure 7 (panel a), which shows the spectra obtained for the eutectic composition, i.e., the mixture containing ENZ + 75 wt % acMAL.

**Figure 7:**
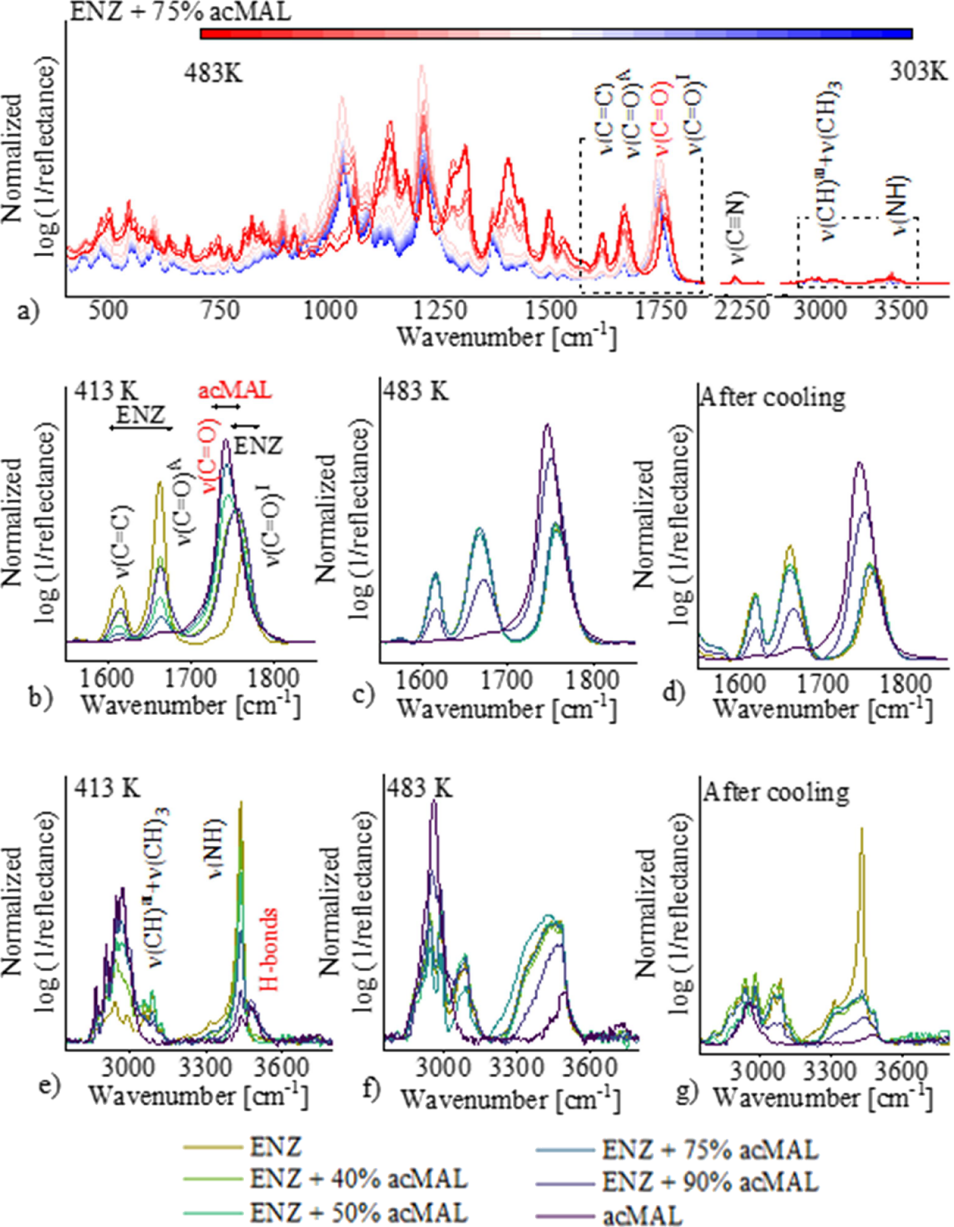
(a) Temperature-dependent infrared spectra of an ENZ + 75 wt % acMAL mixture. Expanded view of (b–d) the imidazolidine and amide-related region (1550–1850 cm^–1^), as well as (e–g) the CH*_x_*, NH, and hydrogen-bonding region (2750–3800 cm^–1^) recorded before melting (413 K), after melting, and at room temperature after cooling the melt. Vibration characteristics of ENZ are shown in black, and those of acMAL are in red.

In the crystalline state, acMAL exhibits weak O–H stretching vibrations in the 3700–3400 cm^–1^ region, arising from lattice water and intramolecular O–H···O contacts, along with CH_2_ stretching bands (2950–2850 cm^–1^), and a strong ester C=O stretching band centered at 1739 cm.^64^ Crystalline ENZ forms inversion dimers through N–H···S, C–H···O, C–H···F, and C–H···N interactions that extend into one-dimensional hydrogen-bonded chains, stabilized further by π···π stacking between trifluoromethyl phenyl rings.^64^ Its characteristic infrared spectrum displays N–H stretching at 3429 cm^–1^, aromatic C–H vibrations (3150–3050 cm^–1^), aliphatic C–H bands (3050–2850 cm^–1^), a sharp nitrile stretch at 2231 cm^–1^, and two carbonyl-related bands at 1765 cm^–1^ (imidazolidine) and 1660 cm^–1^ (amide). Since the characteristic absorption bands of both ENZ and acMAL appear within the ∼1550–1850 cm^–1^ and ∼2150–3750 cm^–1^ regions—which are subtly marked with dashed rectangles in Figure 7a—these spectral ranges were selected for detailed comparative analysis. To assess the nature and evolution of molecular interactions, spectra were analyzed for three distinct physical states of the materials: the crystalline state (measured at *T* = 413 K, below the solidus line—see panels b and e), the isotropic liquid state (at *T* = 483 K, above the liquidus—see panels c and f), and the glassy state obtained by melt-quenching and analyzed at room temperature (*T* < *T*_g_), as labeled “after cooling” in panels b and g.

To elucidate subtle interaction effects between the ENZ and acMAL, second-derivative analysis of the FTIR spectra was performed in the fingerprint region (1580–1850 cm^–1^), allowing precise resolution of overlapping vibrations and detection of composition-dependent perturbations in the carbonyl domain (Figure 7b). The amide C=O stretching vibration of crystalline ENZ, typically centered at 1660 cm^–1^, exhibits a reproducible upshift of approximately 2 cm^–1^ with increasing acMAL content, indicating a mild weakening of the intramolecular N–H···S dimeric associations. This effect is most plausibly attributed to the emergence of weak but competitive dipole–dipole and C–H···O interactions between ENZ and the acetylated functionalities of acMAL (Figure S1, SI). More pronounced and composition-dependent spectral evolution occurs in the high-wavenumber carbonyl region associated with the imidazolidine and ester moieties. In the ENZ + 40 wt % acMAL composition, three distinct second-derivative minima appear at approximately 1763, 1755, and 1740 cm^–1^, reflecting the presence of multiple carbonyl environments arising from partial coordination between ENZ’s C=O groups and acMAL’s polar sites. The two higher-wavenumber bands correspond to perturbed ENZ carbonyls engaged in modified intra- or intermolecular dipolar coupling, whereas the lowest-wavenumber band originates from the ester C=O stretching of acMAL. This spectral pattern indicates the coexistence of different local configurations stabilized by a hierarchy of weak hydrogen bonds (C–H···O, C–H···F) and van der Waals interactions, resulting in partial redistribution of electron density within the carbonyl framework. The ENZ + 40 wt % acMAL composition thus represents an intermediate regime where molecular dispersion and intermolecular coupling promote the formation of an energetically stable mixed crystalline network stabilized by cooperative weak interactions. Upon further increasing the acMAL content to 50% and above, the carbonyl profile simplifies progressively: the imidazolidine-related components weaken, leaving two distinct bands at ∼1753 and 1739 cm^–1^, and ultimately only a single acMAL-derived ester vibration remains, shifting slightly to 1737 cm^–1^ in pure acMAL (Figure S1, SI). This monotonic evolution signifies a structural transition from discrete ENZ + acMAL molecular associations to a matrix dominated by acMAL’s extended hydrogen-bonding and dipolar network. Notably, throughout this transformation, the N–H stretching region remains effectively unshifted, confirming the absence of newly formed strong N–H···O hydrogen bonds and highlighting that the interaction mechanism is governed primarily by weak, cooperative dipolar and van der Waals forces rather than classical hydrogen bonding (Figure 7e).

At the isotropic liquid state (*T* = 483 K), the infrared spectra of neat acMAL, neat ENZ, and their binary mixtures containing 40–75 wt % acMAL exhibit nearly identical spectral patterns in both raw and second-derivative spectra (Figure 7c, Figure S1, SI). This spectral convergence demonstrates complete melting and molecular-level mixing of both components, while the absence of distinct acMAL bands suggests the predominance of ENZ-driven organization. The strong spectral similarity between the mixtures and neat ENZ points to the persistence of ENZ homodimeric structures, stabilized through intermolecular amide–amide hydrogen bonding. Correspondingly, the amide C=O band broadens and shifts to 1667 cm^–1^, reflecting increased conformational heterogeneity and weaker hydrogen bonding due to thermal disorder. Simultaneously, the imidazolidine C=O region resolves into two partially separated bands at 1753 and 1765 cm^–1^, indicating the coexistence of differently coordinated carbonyl groups, possibly reflecting local phase separation at the nanoscale. The ester C=O vibration of acMAL remains unresolved within this region, likely masked by overlapping ENZ contributions, suggesting that acMAL molecules predominantly exist within sugar-rich subdomains rather than forming strong heterointeractions with the API. These features collectively indicate that, within the 40–75 wt % acMAL range, the system exhibits a partially segregated, domain-type molecular organization. ENZ–ENZ and acMAL–acMAL associations dominate, while heteromolecular ENZ–acMAL contacts remain limited. Such a configuration reflects a cooperative melting mechanism in which each component retains its preferential hydrogen-bonding motif, resulting in a homogeneous, isotropic liquid stabilized by microphase separation and dynamic hydrogen-bond exchange.

Upon further increasing acMAL content to 90 wt %, distinct mechanistic changes occur. The ENZ amide C=O band shifts upward to 1673 cm^–1^, consistent with the weakening or partial disruption of ENZ–ENZ hydrogen bonds and the emergence of less strongly bound amide environments. Concurrently, a strong ester band appears at 1749 cm^–1^, revealing the dominance of acMAL’s carbonyl with a sugar-controlled liquid matrix. In neat acMAL, this ester band shifts further downward to 1741 cm^–1^, confirming residual API–sugar interactions perturb the local carbonyl environment in the ENZ + 90 wt % acMAL mixture (Figure S1, SI).

The disappearance of the discrete ENZ N–H stretching band and the emergence of a broad, asymmetric absorption in neat ENZ and in nearly every binary mixture composition demonstrate complete restructuring of the hydrogen-bond network toward a dynamically disordered liquid architecture (Figure 7f).

After melt-quenching, FTIR spectra collected in the glassy state provide mechanistic insight into how acetylated maltose (acMAL) enhances the physical stability of amorphous enzalutamide (ENZ), both individually and in co-amorphous systems (Figure 7d, g).

Upon cooling at 5 K/min, neat ENZ readily recrystallizes, as evidenced by the reappearance of sharp crystalline features and low-intensity shifted bands, indicative of a metastable polymorph distinct from the parent crystal. Both raw and second-derivative spectra of amorphous ENZ closely resemble those of the crystalline form, displaying a sharp N–H stretching band at 3431 cm^–1^ and amide C=O and imidazolidine carbonyl bands at 1660, 1756, and 1765 cm^–1^ (Figure 7d,g, Figure S1, SI). These features reveal multiple conformational states or local polymorphic variants, confirming that amorphous ENZ lacks sufficient configurational restriction to prevent nucleation or structural relaxation during cooling.

In contrast, amorphous acMAL exhibits a broad, featureless spectrum typical of a disordered hydrogen-bonded network, characterized by a weak −H···O band near 3486 cm^–1^ and a single ester C=O vibration centered around 1741 cm^–1^. When ENZ and acMAL form co-amorphous mixtures, progressive inclusion of acMAL substantially reorganizes the molecular environment and modifies intermolecular coupling. For ENZ + 90 wt % acMAL, the FTIR profile resembles that of amorphous acMAL, showing a clearly visible and broad N–H band around 3431 cm^–1^ superimposed on the sugar matrix. This behavior suggests that ENZ molecules are predominantly molecularly dispersed or only partially immobilized within the acMAL hydrogen-bond framework, while a minor fraction undergoes localized phase separation or nanocrystalline ordering. The broad amide and ester signals (minima at 1662/1667 cm^–1^ and 1741/1749 cm^–1^) in both raw and derivative spectra indicate a heterogeneous environment dominated by homomolecular acMAL–acMAL and ENZ–ENZ interactions with only limited ENZ–acMAL hydrogen bonding.

Reducing the acMAL fraction to 75 wt % induces a qualitatively distinct structural regime. The reappearance of a much weaker band related to the N–H stretching band in comparison to a binary mixture with a higher sugar content indicates the partial restoration of ENZ-specific self-association. Concomitantly, the amide C=O band downshifts to 1657 cm^–1^, the ester C=O upshifts to 1754 cm^–1^, and the imidazolidine component at 1765 cm^–1^ becomes more distinct. These correlated shifts reveal cooperative ENZ–acMAL interactions, with the amide group participating more strongly in hydrogen bonding with acMAL’s hydroxyl and carbonyl functionalities. Residual ENZ dimers likely persist in nanodomains, creating a hybrid network with coexisting homo- and heteromolecular interactions. For intermediate compositions with 40–50 wt % acMAL, the N–H stretching band becomes weak or indistinct, implying fewer strong amide-based hydrogen bonds. Nevertheless, the fingerprint region maintains the same organizational pattern as in the 75 wt % system, as confirmed by second-derivative spectra, indicating a similar amorphous framework dominated by weaker or more transient interactions. Here, dipolar and van der Waals forces between ENZ and acMAL are sufficient to kinetically suppress recrystallization, even with a diminished hydrogen-bond contribution.

Collectively, these results outline a composition-dependent stabilization mechanism driven by competitive self-association and cross-association. At a high acMAL content, ENZ retains much of its intrinsic homodimeric structure, which tends to reorganize and crystallize upon cooling. Declining the acMAL content progressively disrupts these dimers, replacing them with a hybrid hydrogen-bonding matrix in which ENZ is confined within an acMAL network. Near and above the eutectic composition, acMAL functions as a dynamic immobilizing medium, restricting the mobility of ENZ molecules and inhibiting nucleation. This transition from self-associated to matrix-confined organization accounts for the experimentally observed deviations from the Gordon–Taylor model and provides direct spectroscopic evidence of interaction-driven stabilization. FTIR analysis thus confirms that eutectic compositions achieve the optimal balance between molecular dispersion and hydrogen-bonding strength, ensuring the maximal physical stability of the pharmaceutically relevant amorphous ENZ phase.

### 3.4 Effect of acMAL on the Isothermal Cold Crystallization Behavior of Amorphous ENZ

Literature reports indicate that co-amorphous systems that possess a eutectic concentration may exhibit superior physical stability when compared to other concentrations or the neat components of the system. Therefore, this section focuses on investigations of the influence of acMAL addition on the recrystallization behavior of supercooled ENZ. As demonstrated in the previous sections, the binary ENZ + acMAL system forms a eutectic mixture at a composition of 75 wt % acMAL. Notably, acMAL possesses a *T*_g_ value more than 30 K lower than that of ENZ, which classifies it as a plasticizer in this binary system. As a result, its incorporation significantly reduces the *T*_g_ of ENZ in the mixture, thereby enhancing the molecular mobility. From this perspective, one might expect an accelerated recrystallization of ENZ when co-formulated with acMAL and investigate it under identical thermal conditions. To investigate the physical stability of both neat ENZ and its binary eutectic mixture, isothermal studies at *T* = 413 K were conducted by means of BDS. Furthermore, to verify the hypothesis that the eutectic composition yields the highest stability, analogous measurements were also performed for the ENZ + 50 wt % acMAL and ENZ + 90 wt % acMAL samples. These compositions enable a comparative analysis of the role of eutectic concentration in improving the physical stability of co-amorphous systems. It should be noted that in the case of neat acMAL, such investigations were not feasible due to the extremely fast cold crystallization of the sample. In fact, crystallization begins before the target temperature of 413 K can be reached and stabilized, rendering isothermal measurements impossible under these conditions.

For each measurement, a fresh sample was prepared by melting the material above its melting point, followed by rapid cooling. The observed cold crystallization process was monitored through a decrease in static permittivity, while its progression was analyzed using the normalized dielectric constant (ε′_*N*_), applied as follows:

10 $${\varepsilon^{\prime }}_{N}\left(t\right)=\frac{\varepsilon^{\prime }\left(0\right)-\varepsilon^{\prime }\left(t\right)}{\varepsilon^{\prime }\left(0\right)-\varepsilon^{\prime }\left(\infty \right)}$$

where ε′(0) represents the dielectric constant at the onset of the crystallization process, ε′(*t*) is the value of the dielectric constant at time *t*, and ε′(∞) corresponds to the long-time equilibrium value. The normalized crystallization kinetic curves of neat amorphous ENZ and the investigated binary co-amorphous mixtures are presented in Figure 8.

**Figure 8:**
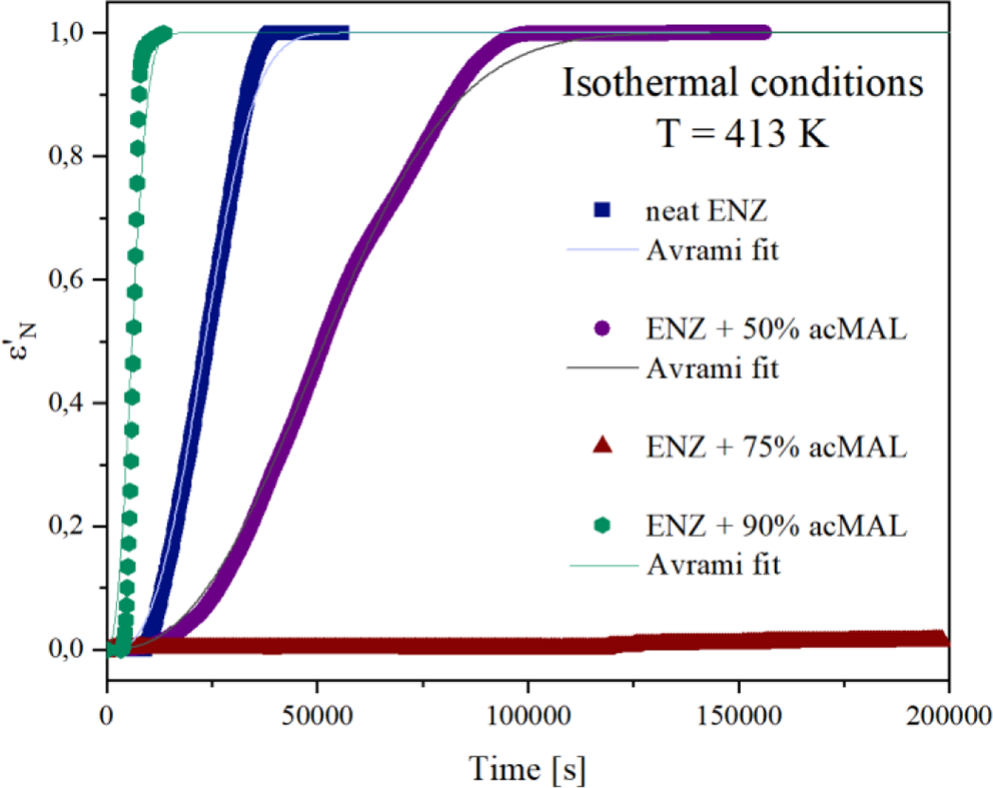
Normalized dielectric constant ε′_*N*_ as a function of time during a cold crystallization process at 413 K of neat ENZ, ENZ + 50 wt % acMAL, ENZ + 75 wt % acMAL, and ENZ + 90 wt % acMAL.

After normalization, a value of 0 corresponds to a fully amorphous system, while an ε′_*N*_ value of 1 indicates the finished crystallization. As can be seen, the co-amorphous ENZ + 90 wt % acMAL undergoes the fastest recrystallization, exhibiting both the earliest onset and end of the recrystallization process. This is due to the presence of a substantial amount of acMAL, which, as mentioned above, starts to recrystallize at much lower temperatures; therefore, the onset of its crystallization could not be captured at *T* = 413 K. The next fastest sample to recrystallize is neat amorphous ENZ. However, it is worth noting that the addition of 50 wt % acMAL leads to a substantial reduction in the crystallization rate of ENZ, with the half-time of crystallization (*t*_0.5_) increasing over 2-fold—from approximately 23 800 s for neat ENZ to 51 600 s for this mixture—proving a notable stabilization effect. Even more pronounced inhibition of recrystallization is observed for the ENZ + 75 wt % acMAL mixture. In this case, the system remains almost entirely amorphous throughout the 55-h duration of the experiment, with no more than 2% crystallization detected. The onset of recrystallization is delayed until approximately 33 h, confirming the remarkable improvement in physical stability of both ENZ and acMAL in their co-amorphous mixture having eutectic concentration. Since the Avrami model is the most commonly used approach for analyzing crystallization kinetics, the kinetic curves presented in Figure 7 were parametrized using this model, which is described by the following formula:^65^

11 $${\varepsilon^{\prime }}_{N}\left(t\right)=1-\exp(-kt{)}^{n}$$

where the constant rate is defined as *k*, and *n* is the Avrami exponent, describing the nucleation dimensionality. Value *n* is usually in the range of 2–3 and depends on the nucleation mechanism and geometric nature of the growing domains.^66^ The values of the fitted parameters are presented in Table 1. The obtained Avrami parameters confirm that the increasing acMAL amount slows the overall crystallization kinetics of ENZ, as indicated by both the lower *n* and the higher *k*. The observed decrease in *n* may suggest a change in the nucleation behavior of the API induced by the presence of acMAL.

**Table 1:** Comparison of the Values of the Constant Crystallization Rate (*K*) and Avrami Exponent (*N*) Obtained from the Modified Avrami Model for Cold Crystallization Kinetics Studies Conducted at 413 K

Sample	*K* [s^–n^]	*N*
Neat ENZ	1.7 × 10^–13^	2.88
ENZ + 50% acMAL	2.5 × 10^–12^	2.43
ENZ + 75% acMAL	-	-
ENZ + 90% acMAL	4.49 × 10^–9^	2.43

It is worth emphasizing once again that the observed improvement in the physical stability of the amorphous ENZ form upon addition of acMAL occurs despite a simultaneous increase in the molecular mobility of the API. To gain deeper insight into this phenomenon, the impact of acMAL on the molecular dynamics of supercooled ENZ was further investigated using BDS, as discussed in the following section.

### 3.5 Effect of acMAL on the Molecular Dynamics of Supercooled ENZ

In this section, the molecular mobility of ENZ + 50 wt % acMAL and ENZ + 75 wt % acMAL was investigated. The primary objective of these experiments was to evaluate the effect of the additive on the molecular dynamics of supercooled ENZ. To compare differences in molecular mobility between the binary amorphous systems, dielectric loss spectra were recorded using BDS. The measurements were carried out over a wide frequency (10^–1^ to 10^6^ Hz) and temperature range from 173.15 to 413.15 K. Representative spectra of the ENZ + 50 wt % acMAL and ENZ + 75 wt % acMAL mixtures are shown in Figure 8, alongside those of the neat components for comparison.

As can be seen, the dielectric loss spectra of all investigated samples exhibit two main features: the dc-conductivity contribution at low frequencies, associated with translational ion motion, and the structural (α) relaxation peak at higher frequencies, related to the cooperative rearrangement of drug molecules. The α-relaxation mode shifts toward higher frequencies with increasing temperature, indicating an enhanced molecular mobility. Upon further heating, the onset of recrystallization is clearly captured in three of the four studied systems, as evidenced by a sudden and significant decrease in the α-relaxation intensity. Based on this behavior, the crystallization onset temperatures were identified as 381 K for neat acMAL and 409 K for both neat ENZ and the ENZ + 50 wt % acMAL mixture. In contrast, the coamorphous system having eutectic concentration (ENZ + 75 wt % acMAL) showed no signs of recrystallization throughout the entire measurement range, further confirming its superior physical stability in the supercooled state. This observation is consistent with previous findings and supports the hypothesis that the coamorphous mixture of ENZ + acMAL at eutectic concentration, due to the strongest specific intermolecular interactions, effectively suppresses recrystallization of ENZ despite the plasticizing effect exerted by the acMAL. For direct comparison of the effect of acMAL on the molecular dynamics of ENZ, one representative spectrum for each sample—recorded at the same temperature (*T* = 373 K)—is highlighted with blue symbols in panels a–d of Figure 9.

**Figure 9:**
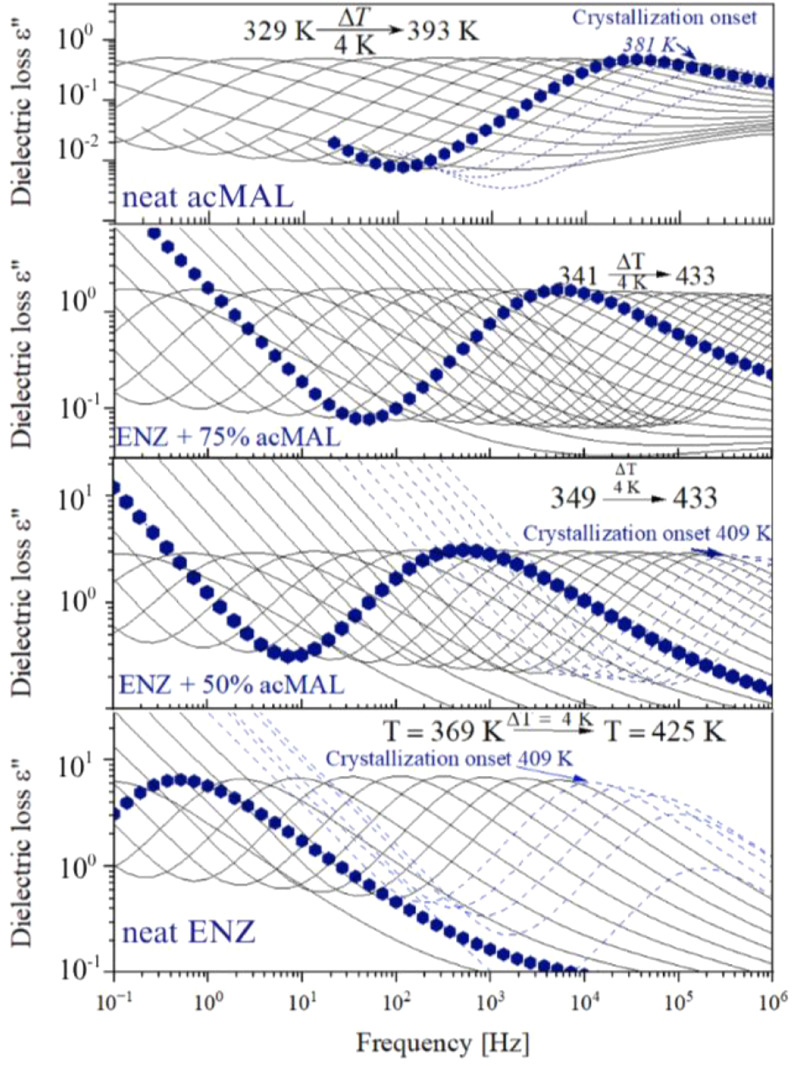
Dielectric spectra of neat ENZ, ENZ + 50 wt % acMAL, ENZ + 75 wt % acMAL, and acetyl maltose (adapted from ref. 26). All spectra were collected above *T*_g_.

Based on a similar analysis of dielectric loss spectra as described previously for neat ENZ, the temperature dependence of the structural relaxation time (τ_α_(*T*)) was determined for the investigated co-amorphous compositions. A comparative plot of τ_α_(*T*) for both the binary systems and the neat components is presented in Figure 10. To parametrize these dependencies, the VFT equation was employed, consistent with the methodology applied in the earlier section. The resulting fits are shown as black lines in Figure 10, and the corresponding VFT parameters are summarized in the table located in the bottom right corner of the figure. Since the VFT model provided a good description of τ_α_(*T*) dependences for all investigated systems, it was further used to estimate their kinetic glass transition temperature and fragility index. The obtained *T*_g_ values are consistent with those determined from the calorimetric method, clearly showing a substantial decrease in the glass transition temperature of ENZ with increasing acMAL content in the mixture. In contrast, *m_p_* increases as the acMAL concentration increases, indicating that the systems become dynamically more fragile. This trend reveals a lack of direct correlation between fragility and physical stability in the studied amorphous systems.

**Figure 10:**
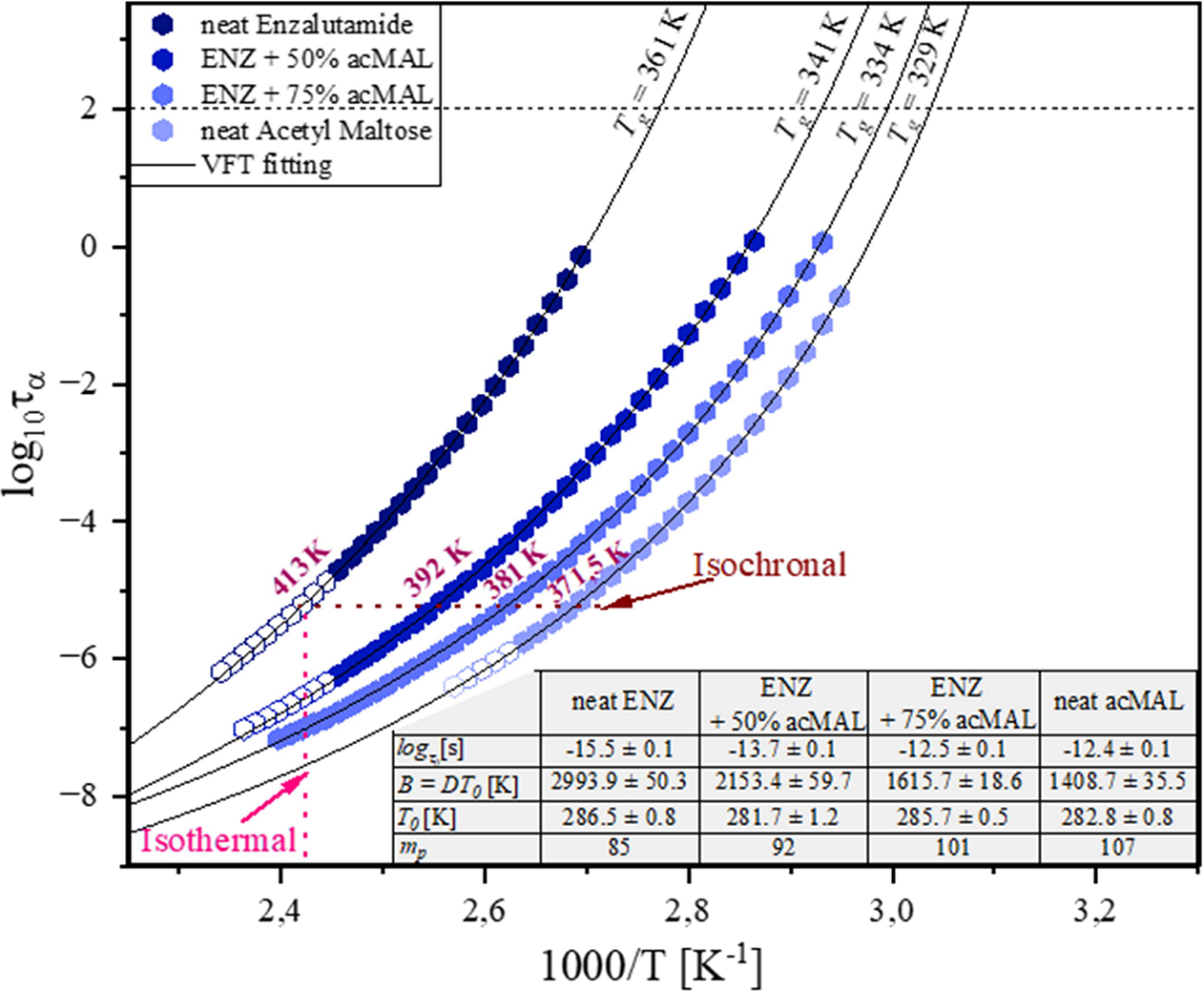
Relaxation map of neat ENZ, ENZ + 50 wt % acMAL, ENZ + 75 wt % acMAL, and neat acMAL. The temperature dependence of τ_α_ in the supercooled liquid has been described by the VFT equation.

To clearly illustrate the extent to which the molecular dynamics of ENZ are accelerated by the presence of acMAL, two reference lines were included in Figure 10. The first corresponds to the isothermal conditions (*T* = 413 K) under which the physical stability experiments were conducted. The second represents an isochronal condition, defined as the temperature at which each system exhibits the same α-relaxation time (τ_α_) as neat ENZ at 413 K. As shown, a significant improvement in the physical stability of ENZ was observed upon addition of acMAL, despite the fact that the measurement temperature (*T* = 413 K) is 21 and 32 K higher than the isochronal reference temperatures for the ENZ + 50 wt % acMAL and ENZ + 75 wt % acMAL mixtures, respectively. This observation highlights that the actual stabilization effect exerted by acMAL is even greater than what is inferred from isothermal experiments alone. Taking into account the remarkable suppression of ENZ recrystallization achieved by acMAL—particularly in its amorphous form and at eutectic concentration—the subsequent part of this study focuses on evaluating whether acMAL can also enhance the supersaturation level of ENZ with comparable efficiency.

### 3.6 Powder Dissolution Studies under Biorelevant Conditions

The ability of acMAL to promote and maintain elevated dissolved drug concentrations was first assessed in simple aqueous media as a preliminary screening experiment. These apparent solubility measurements, performed in purified water using a standardized flask-shaking protocol, are provided in Table S1, SI and serve solely to motivate biorelevant dissolution studies. The obtained results indicate that amorphization itself enhances the apparent solubility of ENZ in the tested 24-h period, as expected for disordered solids. However, a further and more pronounced increase was observed upon addition of acMAL, with the highest apparent solubility recorded for the coamorphous ENZ + 75 wt % acMAL (i.e., corresponding to the eutectic composition). Notably, a further increase in acMAL content (90 wt %) resulted in a slight reduction in measured solubility after 24 h, suggesting that the optimum performance corresponds to the co-amorphous system having a eutectic ratio rather than a simple dilution effect. Such behavior is consistent with previous findings for eutectic and co-amorphous systems.^67^

To verify whether the trends observed in water persist under more physiologically relevant conditions, powder dissolution tests were performed under non-sink conditions in Fasted State Simulated Intestinal Fluid (FaSSIF, pH 6.5). Since polymeric carriers such as hydroxypropyl methylcellulose acetate succinate (HPMCAS) are commonly employed in marketed amorphous solid dispersions, this excipient was also incorporated into the tested formulations for comparative purposes. The inclusion of HPMCAS enabled the assessment of how the proportion of acetylated maltose (acMAL) affects the release behavior of ENZ in the presence of a polymeric matrix representative of commercial ASD systems. Accordingly, powder dissolution studies were carried out under non-sink conditions in FaSSIF with a fixed 40 wt % HPMCAS, while varying the ENZ-to-acMAL ratio to investigate the effect of acMAL concentration (see Table 2). The total mass of each formulation was 300 mg. Maintaining constant polymer content ensured that differences in dissolution performance reflected the intrinsic effect of acMAL rather than variations in polymer concentration.

**Table 2:** Compositions of the Tested Formulations

Formulation	ENZ [mg]	acMAL [mg]	HPMCAS [mg]
ENZ + 40% HPMCAS	**180**	0	120
(ENZ + 50% acMAL) + 40% HPMCAS	**90**	90	120
(ENZ + 75% acMAL) + 40% HPMCAS	**45**	135	120
(ENZ + 90% acMAL) + 40% HPMCAS	**18**	162	120

As shown in Figure 11a, all tested formulations exceeded the equilibrium concentration of neat ENZ in FaSSIF (5.82 ± 0.17 μg/mL), determined experimentally in separate solubility measurements (24–48 h plateau under identical medium and temperature conditions, described in Section 2). This indicates that supersaturation was generated in each case, a key factor that enhances the bioavailability of poorly soluble compounds such as ENZ.^67^ However, differences in the temporal evolution of the dissolution profiles were observed, depending on drug loading and acMAL content. Formulations containing higher ENZ content (0% and 50% acMAL) reached higher concentrations initially, but after 120–150 min, the concentration reached a plateau, while acMAL-rich formulations (75% and 90%) exhibited slower initial release but more gradual, continuously increasing dissolution profiles over the entire duration of the experiment.

**Figure 11:**
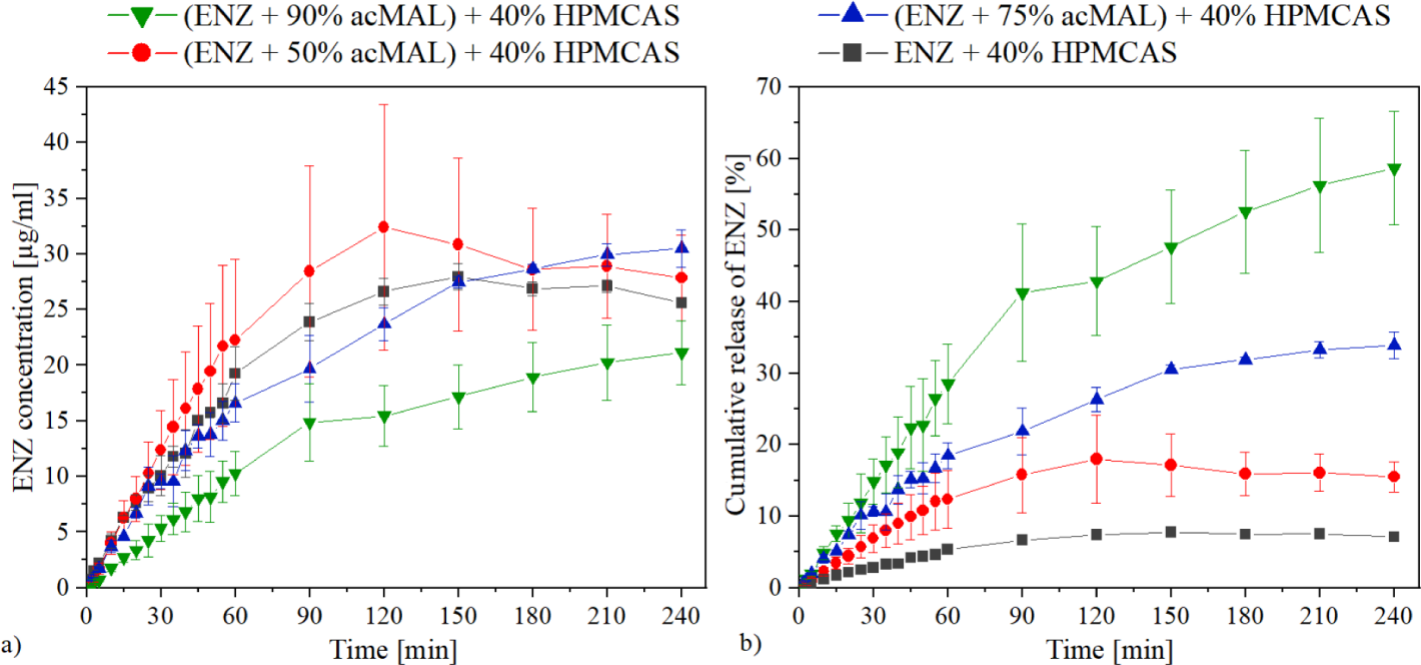
Nonsink powder dissolution profiles of ENZ formulation in FaSSIF (pH 6.5) at 310 K, with a paddle speed of 50 rpm. Panel (a) shows the absolute ENZ concentrations, whereas panel (b) presents the dose-normalized cumulative ENZ release.

To enable a meaningful comparison of dissolution performance across formulations with different drug loadings, the dissolved ENZ was normalized to the loaded dose, as shown in Figure 11b. When expressed in this manner, clear and systematic differences between the formulations become evident. The formulation containing ENZ without acMAL exhibits the lowest released fraction over the entire dissolution period, indicating limited release efficiency despite the highest absolute drug load. Among the tested systems, the formulation containing 90 wt % acMAL achieves the highest cumulative released fraction at all investigated time points. This observation primarily reflects the lower initial ENZ loading combined with a higher relative fraction of ENZ released under nonsink conditions. The eutectic composition (ENZ + 75 wt % acMAL) represents an intermediate but particularly relevant case. Although it contains substantially less ENZ than the ENZ-rich formulations, it achieves a high released fraction combined with a stable and progressively increasing release profile. Overall, Figure 11b demonstrates that increasing the acMAL content systematically improves the efficiency and sustainability of ENZ release when evaluated on a dose-normalized basis. It should be emphasized that this study focuses on solution-phase behavior. The solid-state form of any precipitate was not determined here, and thus no assumptions are made regarding whether ENZ reprecipitated as crystalline or amorphous. A dedicated analysis of the precipitated phase will be pursued in future work.

Taken together, these findings underscore the importance of evaluating dissolution performance using both absolute concentrations and dose-normalized metrics, particularly when comparing formulations with different drug loadings. The results further demonstrate that trends observed in simple aqueous media translate, in a qualitative manner, to biorelevant conditions, while emphasizing that dissolution behavior under non-sink conditions reflects a balance between formulation composition, drug loading, and solution-phase processes.

## 4 Conclusions

This study demonstrated that the ENZ + acMAL binary system forms a eutectic composition at 75 wt % acMAL, with the eutectic point in agreement with the Schröder-van Laar prediction, although the experimentally determined liquidus lines show pronounced deviations from the theoretical model. This deviation suggests non-ideal mixing behavior in the crystalline phase, likely arising from specific intermolecular interactions that disrupt simple thermodynamic predictions. In subsequent investigations, attention was focused on the amorphous forms of the ENZ + acMAL systems, which were prepared via melt-quenching. Analysis of DSC thermograms of co-amorphous systems revealed substantial deviations of the glass transition temperature (*T*_g_) from Gordon–Taylor predictions, indicating strong heteromolecular interactions, which were further supported by FTIR results. It needs to be pointed out that the *T*_g_ of ENZ significantly decreased upon addition of acMAL, which, in light of the plasticizing effect, would typically suggest a decrease in physical stability. Despite the pronounced plasticizing effect of acMAL on ENZ, both calorimetric and infrared as well as dielectric studies reveal inhibition of ENZ’s recrystallization in the presence of the employed additive. Co-amorphous composition with eutectic concentration exhibited exceptional physical stability under isothermal conditions (*T* = 413 K), delaying the onset of recrystallization of neat ENZ by over 30 h and limiting its crystallinity to ≤2% after 55 h. Broadband dielectric spectroscopy (BDS), in addition to assessing physical stability, also provided further insight into the molecular mobility of the studied systems, enabling direct comparison between the eutectic and non-eutectic compositions. In this work, the molecular dynamics of neat amorphous ENZ were also characterized for the first time, both in the supercooled liquid and glassy states, establishing a critical reference for evaluating the impact of acMAL. While the apparent solubility study indicated that the amorphous eutectic composition (ENZ + 75 wt % acMAL) exhibited the highest concentration after 24 h, this result most likely reflects solution-phase behavior under the applied experimental conditions and does not by itself establish a direct mechanistic link to solid-state stability. In the subsequent dissolution tests, both the eutectic and 90 wt % acMAL systems maintained elevated dissolved concentrations for at least 4 h, whereas the remaining formulations reached a plateau in dissolved drug concentration after approximately 120–150 h. Notably, the eutectic composition achieved dissolved concentrations nearly identical to those of the higher-acMAL formulation despite its lower drug loading, indicating an efficient utilization of the loaded drug that cannot be explained by excipient dilution alone.

Overall, the results confirm that converting eutectic composition in coamorphous systems offers a powerful formulation strategy to simultaneously enhance amorphous physical stability and favorable dissolution behavior with potential applicability to other poorly water-soluble APIs. Furthermore, this work underscores the importance of integrating phase diagram analysis, molecular mobility measurements, and dissolution testing to fully understand the performance of co-amorphous drug systems. The findings also suggest that the eutectic-driven stabilization mechanism identified here may be transferable to other API-excipient pairs, opening new avenues for the design of advanced solid-state formulations.

## Supplementary Material



## Notes

Additional FTIR analyses of ENZ–acMAL systems, presenting second-derivative spectra for crystalline, molten, and quenched amorphous samples, which provide detailed insight into composition-dependent intermolecular interactions; and preliminary apparent solubility data obtained using the shake-flask method, comparing crystalline and amorphous ENZ and its coamorphous formulations with varying acMAL content (PDF)
