# Supplementary material for: Eutectic Coamorphous System of Enzalutamide and Acetyl Maltose: A Strategy for Improved Physical Stability and Aqueous Solubility
Source: Mol Pharm. 2026 Feb 23;23(4):2302–18. doi: 10.1021/acs.molpharmaceut.5c01164 (PMC13318067; doi:10.1021/acs.molpharmaceut.5c01164)
Supplement: Supplementary file 1 [file mp5c01164_si_001.pdf]

Eutectic co-amorphous system of enzalutamide and acetyl maltose: a strategy for improved physical stability and aqueous solubility

Julia Cichocka-Łokuciejewska[1,2]*, Justyna Knapik-Kowalczuk[1]*, Mateusz Dulski[3], Katarzyna Greber[2], Wiesław Sawicki[2], Marian Paluch[1]

[1]Institute of Physics, University of Silesia in Katowice, 75 Pułku Piechoty 1A, 41-500, Chorzów, Poland

[2]Department of Physical Chemistry, Faculty of Pharmacy, Medical University of Gdańsk, Al. Gen. J. Hallera 107, 80-416, Gdańsk, Poland

[3]Institute of Materials Science, University of Silesia in Katowice, 75 Pułku Piechoty 1A, 41-500, Chorzów, Poland

*e-mail: justyna.knapik-kowalczuk@us.edu.pl, julia.cichocka@gumed.edu.pl

Supporting information

This section corresponds to Subsection 3.3. of the main manuscript.

Investigation of intermolecular interactions in ENZ + acMAL mixtures using FTIR spectroscopy

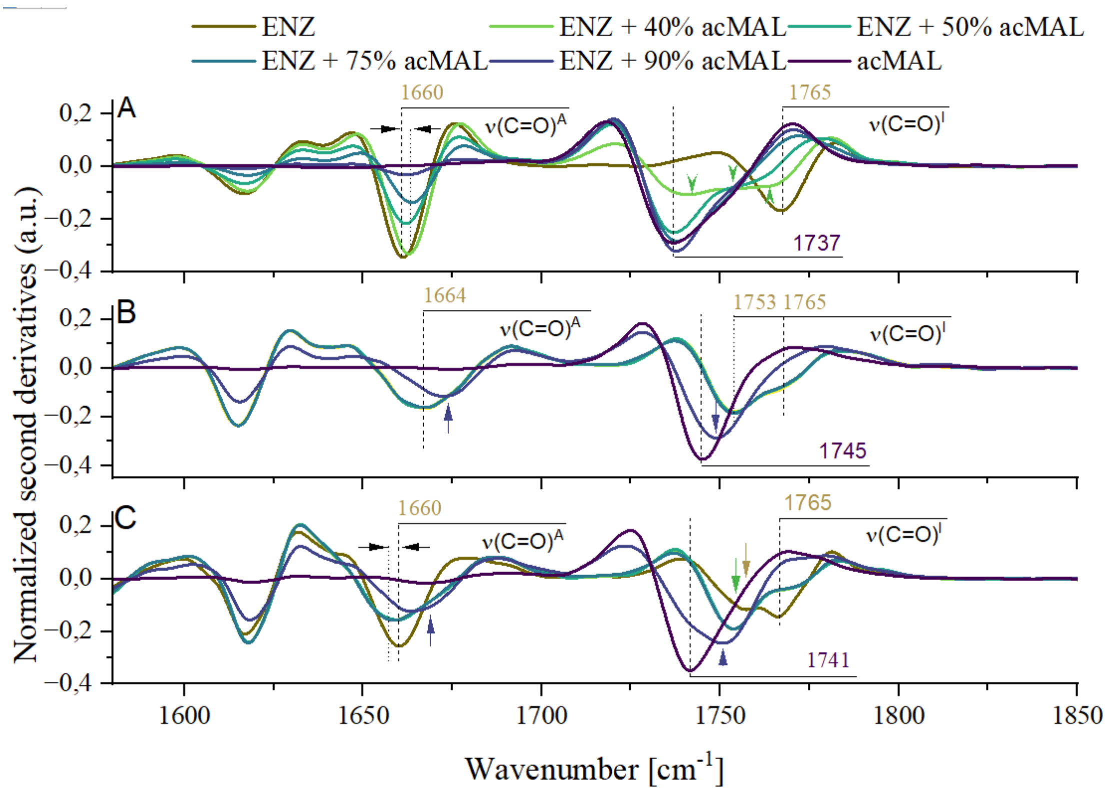

Figure S1. Second-derivative FTIR spectra in the fingerprint region (1580–1850 cm^{-1}) are presented for (A) the crystalline systems, (B) the fully melted mixtures, and (C) the quenched amorphous systems. Arrows indicate spectral bands corresponding to molecular environments distinct from those of the pure API and pure sugar components.

This section corresponds to Subsection 3.6.1. of the main manuscript.

Apparent solubility in water (preliminary assessment)

Experimental Method

A so called shake-flask method was used to study the apparent solubility of pure API, in both crystalline and amorphous forms, as well as amorphous binary mixtures with 50 wt.%, 75 wt.% and 90 wt.% of acMAL. For this purpose, 3 ml of purified water was added to an excess of tested substances (2 mg). Then the samples were shaken in a water bath at 298.15 K for 24 hours. After that, the samples were filtered using a 0.45 μm syringe filter to remove particulate matter and analyzed as described in section 2.5.2.

Results

The initial evaluation of the effect of acMAL on the apparent concentration in water of ENZ was performed using a so-called flask-shaking method. An excess of the solid sample was putted in purified water at 298 K for 24 h under continuous agitation. This simple and standardized procedure, commonly used for early solubility screening, was applied as the first step in assessing the influence of acMAL on the apparent solubility of ENZ, and to verify its ability to maintain supersaturation. Both crystalline and amorphous ENZ were examined, along with their binary physical mixtures and the corresponding co-amorphous systems containing 50, 75, and 90 wt.% of acMAL. Such an experimental design allowed differentiation between the effects resulting purely from amorphization and those additionally induced by molecular interactions between ENZ and acMAL. After 24h of shaking, the suspensions were filtered and analyzed by HPLC and quantified against a previously validated calibration curve. The obtained results are summarized in Table S1.

Table S1. Apparent water solubility of ENZ and ENZ-based formulations in both, crystalline and amorphous states after 24h shaking period in 298 K.

**Composition**	**ENZ + acMAL ratio [wt.%]**	ENZ apparent **solubility in water [μg/mL]**	**Relative increase vs. neat ENZ (after 24 h)**
**Neat ENZ (cryst.)**	100:0	2.20 ± 0,72	**-**
**Neat ENZ (amorf.)**	100:0	4.63 ± 1,87	**110.45 %**
**ENZ + 50 wt.% acMAL (cryst.)**	50:50	3.60 ± 1,25	**63.64 %**
**ENZ + 50 wt.% acMAL (amorf.)**	50:50	4.93 ± 1,88	**124.09 %**
**ENZ + 75 wt.% acMAL (cryst.)**	25:75	3.05 ± 1,26	**38.64 %**
**ENZ + 75 wt.% acMAL (amorf.)**	25:75	7.57 ± 2,33	**244.09 %**
**ENZ + 90 wt.% acMAL (cryst.)**	10:90	1.98 ± 0,07	**-9.90 %**
**ENZ + 90 wt.% acMAL (amorf.)**	10:90	3.56 ± 0,40	**61.82 %**

As can be seen the results indicate that amorphization itself enhances the apparent solubility of ENZ in the tested 24h period, as expected for disordered solids. However, a further and more pronounced increase was observed upon addition of acMAL, with the highest apparent solubility recorded for the co-amorphous ENZ + 75 wt.% acMAL (i.e., corresponding to the eutectic composition). Notably, a further increase of acMAL content (90 wt.%) resulted in a slight reduction in measured solubility after 24h, suggesting that the optimum performance corresponds to the co-amorphous system having an eutectic ratio rather than a simple dilution effect. Such a behavior is consistent with previous findings for eutectic and co-amorphous systems[72]. Although improved wettability may accelerate the initial dissolution process, the enhanced apparent concentration of ENZ in the presence of acMAL is more likely attributed to molecular-level interactions between ENZ and acMAL, confirmed by FTIR and DSC analysis and the highest physical stability of the eutectic mixture, as shown in BDS study. These interactions, combined with the hydrophilic nature of acMAL, can promote the formation of mixed water–acMAL solvation clusters around ENZ molecules, enhancing their dispersion and apparent concentration in aqueous media. These preliminary findings suggest that the ENZ + acMAL co-amorphous systems, particularly the one corresponding to the eutectic composition, exhibit improved aqueous solubility performance compared to both crystalline and amorphous ENZ alone. Consequently, the water-based test served as a preliminary screening tool, confirming the potential of acMAL as a supersaturation stabilizer, and therefore apparent solubility-enhancing compound and justified further evaluation under biorelevant dissolution conditions.

[72] França, M. T.; Martins Marcos, T.; Costa, P. F. A.; Bazzo, G. C.; Nicolay Pereira, R.; Gerola, A. P.; Stulzer, H. K. Eutectic Mixture and Amorphous Solid Dispersion: Two Different Supersaturating Drug Delivery System Strategies to Improve Griseofulvin Release Using Saccharin. *Int. J. Pharm.* **2022**, *615*, 121498. https://doi.org/10.1016/j.ijpharm.2022.121498.
